# A Microwell Array Embedded Microfluidic Gradient Platform for Drug Screening on Tumor Spheroids

**DOI:** 10.1002/smll.202514775

**Published:** 2026-04-21

**Authors:** Ling Liu, Guoying Wang, Ming Li

**Affiliations:** ^1^ School of Engineering Macquarie University Sydney NSW Australia; ^2^ School of Mechanical and Manufacturing Engineering The University of New South Wales Sydney NSW Australia; ^3^ Macquarie Medical School Faculty of Medicine Health and Human Sciences Macquarie University Sydney NSW Australia

**Keywords:** co‐culture spheroid, high‐throughput drug evaluation, microfluidic gradient platforms, microwell, tumor spheroid

## Abstract

Microfluidic gradient platforms, especially flow‐based types, have emerged as promising tools in 3D tumor spheroid research, as they enable precise control of concentration gradients with rapid establishment and sustained long‐term stability. However, current flow‐based gradient chips are often constrained by limited spheroid culture capacity, restricting their utility for large‐scale drug evaluation. Here, we developed a microfluidic gradient platform embedded with high‐density microwell arrays (MEG platform), which enables high‐throughput and physiologically relevant spheroid culture across multiple chambers on a single chip. The total number of spheroids is flexibly tuned by adjusting the microwell array sheet size, and both monoculture and co‐culture spheroids maintain continuous and stable growth with well‐defined morphology. The platform demonstrates stable gradient delivery under dynamic perfusion. It is further applied to assess drug responses in monoculture and co‐culture spheroids, with co‐cultures exhibiting enhanced resistance compared with monocultures. The high‐throughput MEG platform should facilitate the use of tumor spheroid models in large‐scale drug testing and tumor‐stromal interaction studies.

## Graphical abstract

A microfluidic gradient platform embedded with high‐density microwell arrays (MEG platform) enables high‐throughput generation of uniform tumor spheroids for gradient‐based drug response assessment under dynamic perfusion. As a proof of concept, monoculture breast cancer cell (MCF‐7) spheroids and MCF‐7/human dermal fibroblast (HDF) co‐culture spheroids were investigated, revealing distinct morphological characteristics and differences in therapeutic responses between the two models.

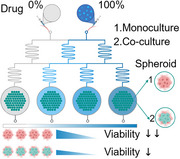

## 1 Introduction

3D tumor spheroids have emerged as critical in vitro models in cancer research, particularly for drug discovery and development [1, 2]. Compared with traditional 2D cultures, spheroids better recapitulate in vivo tissue architecture, cellular heterogeneity, and diffusion‐limited microenvironments, making them more physiologically relevant for studying tumor biology and therapeutic responses [3, 4]. As interest in tumor spheroid‐based studies grows, there has been an increasing demand for platforms that enable precisely control drug exposure and concentration gradients. Microfluidic gradient platforms are particularly promising in this context, offering spatiotemporal regulation of biomolecular gradients, high reproducibility, and real‐time tunability [5, 6, 7]. Such features are essential for investigating gradient‐mediated cellular processes and dose‐response relationships.

Microfluidic gradient platforms have been developed in various formats, including flow‐based, diffusion‐based, and droplet‐based systems [8, 9, 10]. Among these, flow‐based microfluidic gradient platforms leverage laminar flow and molecular diffusion at the microscale to generate reproducible gradients with rapid response times and controllable profiles [8, 11]. Unlike conventional stepwise addition methods, flow‐based systems provide continuous, real‐time, and tunable concentration landscapes within a single, uniform microenvironment [12, 13, 14]. These platforms have been widely used to investigate tumor microenvironmental cues, drug efficacy, and toxicity in tumor spheroids [7, 15, 16, 17, 18]. Despite these advantages, most existing flow‐based microfluidic gradient platforms support only a limited number of spheroids per device [19], restricting throughput and reducing statistical robustness. Furthermore, efficient cell loading [20, 21, 22, 23] and stable long‐term perfusion [24, 25] remain major challenges, limiting their broader application in tumor spheroid assays.

To address these limitations, we developed a microfluidic gradient platform embedded with high‐density microwell arrays (MEG platform). This platform enables the parallel formation of hundreds of uniform spheroids across multiple culture chambers on a single chip. We characterized spheroid growth and morphological changes in both monoculture and co‐culture settings, validated stable gradient delivery, and applied the platform to evaluate drug responses. By combining precise gradient control with high‐density microwell arrays, this platform facilitates physiologically relevant, high‐throughput tumor spheroid assays under dynamic perfusion, providing a valuable tool for drug screening, therapeutic evaluation, and modeling tumor‐stromal interactions.

## 2 Results

### 2.1 Establishment and Characterization of the MEG Platform

**FIGURE 1:**
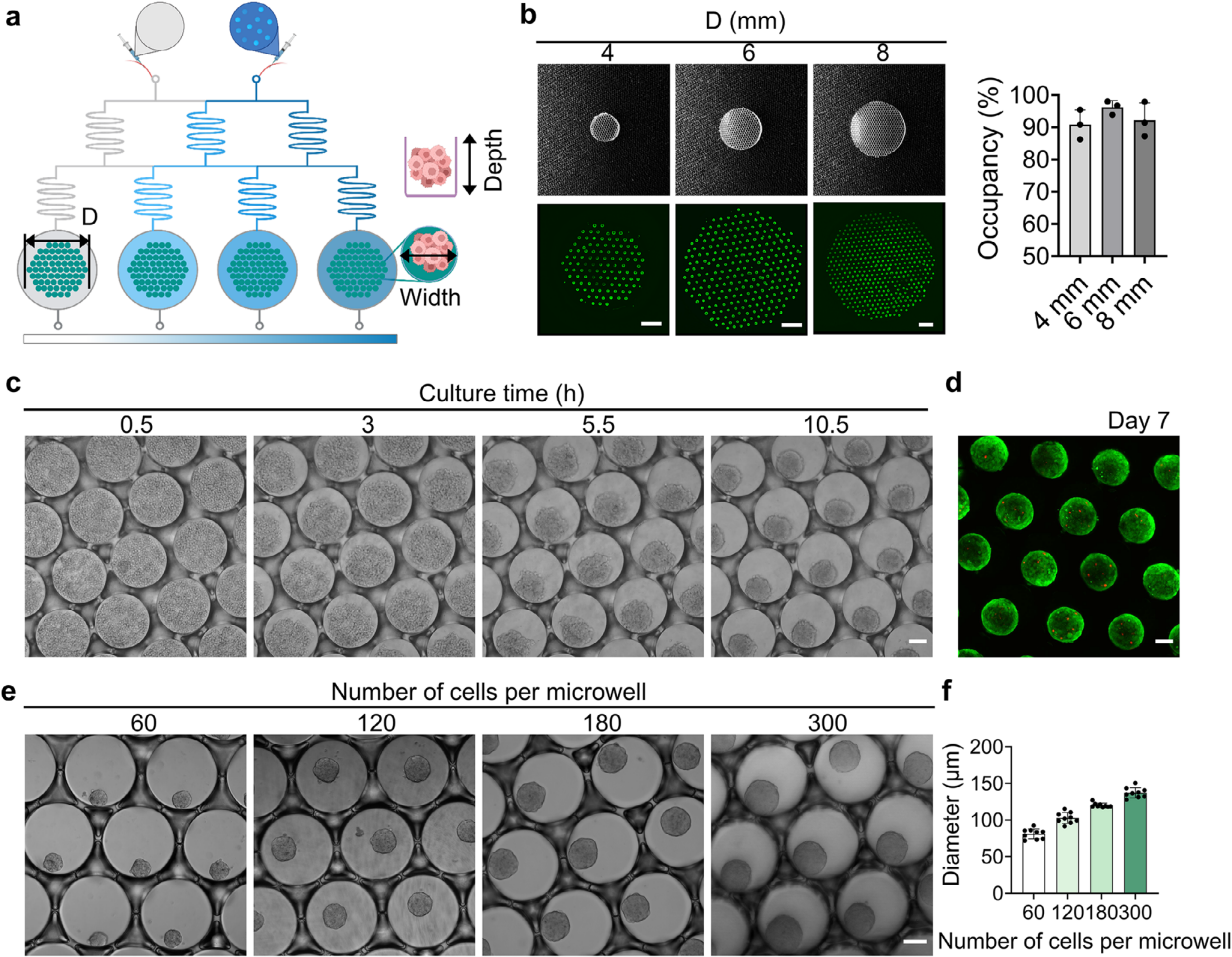
MEG platform for high‐throughput tumor spheroid culture and drug testing. (a) Overview schematic of the gradient generator with two inlets connected to four culture chambers embedded with microwell arrays. Microwell dimensions: 300 µm × 300 µm (width × depth). (b) Size‐tunable microwell array sheets (4, 6, 8 mm) and seeding performance. Top: Representative images of microwell array sheets. Bottom: Representative fluorescence images of spheroids stained with Calcein‐AM. D denotes the diameter of the microwell array sheet. Right: Quantitative analysis of seeding efficiency by sheet size. (c) Bright‐field images showing staged aggregation of MCF‐7 cells into compact spheroids on microwell array sheets. (d) Spheroid morphology and viability after 7‐day medium perfusion, stained with Calcein‐AM (C‐AM; live, green) and Ethidium homodimer‐1 (EthD‐1; dead, red). (e) Representative spheroid images and (f) quantitative analysis of spheroid diameters formed from different initial cell numbers (60, 120, 180, 300 cells per microwell). Scale bars: (b) 1 mm; (c, d, e) 100 µm. Data are presented as mean ± SD (n = 3 independent biological replicates). In (b), each data point represents spheroid occupancy from one microwell array sheet. In (f), each dot represents the average spheroid diameter in one chamber (three chambers per condition).

We developed an MEG platform that enables stable, parallel, and dynamic drug treatment of monoculture and co‐culture tumor spheroids, while preserving the high‐throughput capacity and size‐controllability of microwell systems. The key structure of MEG platform is a Christmas tree gradient generator and cell culture chambers loading microwell array sheets, both fabricated using standard soft lithography. The concentration gradient generator is based on a serpentine channel design, which extends the effective flow path within a limited space and thereby enhances the stability of branched fluid streams. The gradient generator was connected to four cell culture chambers, each accommodating a microwell array sheet to support tumor spheroid culture (Figure 1a). The microwells were designed with a dimension of 300 µm in both diameter and depth. The 300 µm × 300 µm microwell geometry was selected to balance stable spheroid formation, efficient mass transport, and fabrication feasibility [26, 27, 28]. In this study, the microwells had a fixed diameter of 300 µm, while the overall size of the microwell array sheet could be adjusted according to different experimental requirements. Figure 1b shows representative microwell array sheets with diameters of 4, 6, and 8 mm, containing approximately 110, 245, and 435 microwells, respectively. In this study, the mean spheroid occupancy rates of these sheets reached 90.8%, 96.2%, and 92.3%, demonstrating the reliability and scalability of spheroid formation.

We first used MCF‐7 cells to evaluate the performance of MEG platform (Figure 1c). After cell seeding on the MEG chip, MCF‐7 cells rapidly aggregated within 30 min, accompanied by a marked decrease in projected area. After 5.5 h, all MCF‐7 cells compacted into 3D aggregates with irregular shape. The round MCF‐7 spheroids were formed after 10.5 h incubation. This rapid cell aggregation and growth on the platform demonstrated its biocompatibility and its capacity to provide a supportive microenvironment for reliable 3D spheroid culture. To assess the impact of long‐term perfusion on spheroid integrity and survival, culture medium alone was perfused for 7 days through the chambers, and spheroid viability was evaluated by live/dead staining. Spheroids maintained intact morphology and were predominantly composed of viable cells under perfusion (Figure 1d). These results indicate that, within the flow rate range used, perfusion exerted minimal influence on viability, confirming the suitability of the platform for dynamic culture and treatment.

By varying the initial seeding density, we achieved precise control over the size of spheroids formed on the platform. MCF‐7 cells seeded at densities of 60, 120, 180, and 300 cells per microwell formed healthy spheroids with smooth edges and compact structures (Figure 1e). Quantitative analysis (Figure 1f) showed that spheroid diameter increased with initial seeding density, with mean diameters ranging from 81.1 µm at 60 cells per microwell to 137.2 µm at 300 cells per microwell. All groups exhibited narrow size distributions on Day 1.

Spheroid formation efficiency was quantitatively evaluated at different seeding densities (see Figure S3). The size uniformity of spheroids both within and between chambers was quantitatively assessed (see Figure S4). These results validate the high‐throughput capability and excellent reproducibility of the platform for generating uniform spheroids. In addition, confocal Z‐stack imaging revealed comparable thickness and diameter, confirming a nearly spherical 3D architecture with high structural homogeneity (Figure S5). Furthermore, the platform is not limited to MCF‐7 cells and can also support the formation of 3D spheroids from other cell lines (see Figure S6), highlighting its broad applicability.

### 2.2 Co‐Culture Spheroids on the MEG Platform

Complex physical and chemical interactions between cancer cells and stromal cells (e.g., fibroblasts) play a pivotal role in regulating cancer progression, metastasis, and resistance to anticancer therapies [29, 30]. Therefore, it is essential to establish a robust and physiologically relevant in vitro model that supports multicell co‐culture, precise size control, and stable drug delivery for reliable drug screening and testing.

**FIGURE 2:**
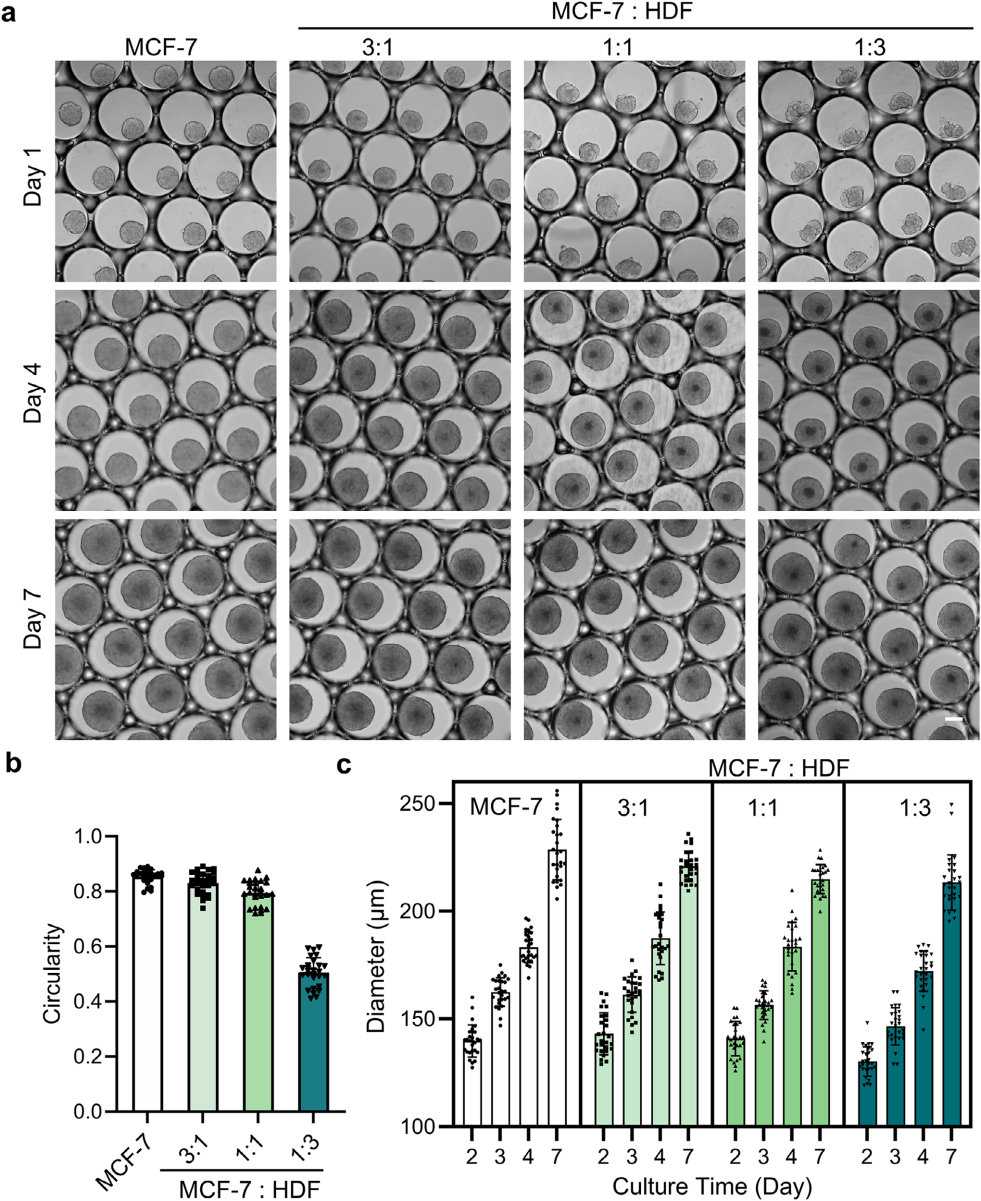
Morphogenesis and growth of monoculture (MCF‐7) and co‐culture (MCF‐7/HDF) spheroids on the MEG platform. (a) Representative bright‐field images of MCF‐7 monoculture spheroids and MCF‐7: HDF co‐culture spheroids at 3:1, 1:1, and 1:3 seeding ratios on Days 1, 4, and 7. (b) Quantitative analysis of spheroid circularity (calculated as 4πA/P^2^) on Day 1 for the four seeding groups. (c) Quantitative analysis of spheroid growth over time for the four seeding groups. Scale bar: 100 µm. Data are presented as mean ± SD (n = 3 independent biological replicates). In (b) and (c), individual data points represent individual spheroids (technical replicates).

To validate the applicability of the platform for multicellular co‐culture, we first established tumor cell/fibroblast co‐culture spheroids on the MEG platform. Specifically, MCF‐7 cells and human dermal fibroblasts (HDFs) were co‐seeded at ratios of 3:1, 1:1, and 1:3, while keeping the total cell number constant across groups. Figure 2a shows the morphology of MCF‐7 monoculture spheroids and MCF‐7/HDF co‐culture spheroids at different seeding ratios (3:1, 1:1, 1:3) on Days 1, 4, and 7. On Day 1, smooth and compact spheroids were observed in most groups, while the 1:3 co‐culture group formed less spherical aggregates with irregular boundaries. Quantitative circularity analysis confirmed this observation, showing notably lower circularity in the 1:3 group compared with the others (Figure 2b). By Day 2, spheroids in the 1:3 co‐culture condition had compacted into round morphologies, resembling those in the other co‐culture conditions. Representative Day 2 images of the MCF‐7/HDF (1:3) co‐culture and MCF‐7 monoculture control are shown in Figure S7a. A similar transition, from irregular aggregates to round spheroids, was also observed in monoculture HDF spheroids (Figure S7b). This indicates that the early irregularity in the HDF‐rich co‐culture condition is likely attributable to the intrinsic aggregation behavior of HDFs. The diameters of both monoculture and co‐culture spheroids (at different seeding ratios) increased over time (Figure 2c). By Day 7, monoculture MCF‐7 spheroids reached an average diameter of 228.6 µm, whereas spheroids from the 3:1, 1:1, and 1:3 co‐culture groups measured 220.9, 214.9, and 213.4 µm, respectively, indicating a trend toward smaller sizes in co‐culture conditions.

**FIGURE 3:**
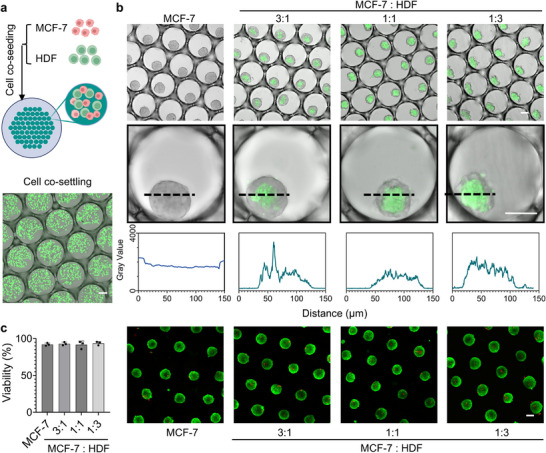
Spatial distribution of HDF and viability in co‐culture spheroids. (a) Schematic of HDF pre‐labeling with C‐AM and seeding into microwells, together with a representative image showing mixed MCF‐7 cells and HDFs settled at the microwell bottom. (b) Representative fluorescence image at 16 h showing central localization of C‐AM‐labeled HDFs. The boxed spheroid is magnified for line‐scan analysis, yielding a center‐peaked fluorescence profile confirming HDF core localization. (c) Live/dead staining (C‐AM/EthD‐1) on Day 2 and quantitative analysis of spheroid viability from MCF‐7 monocultures and MCF‐7: HDF co‐cultures at 3:1, 1:1, and 1:3 ratios. Scale bars: 100 µm. Data are presented as mean ± SD (n = 3 independent biological replicates). Each data point represents the mean viability of multiple spheroids (technical replicates).

To further understand the distribution of HDFs in co‐culture spheroids, we pre‐stained the HDFs with Calcein‐AM (C‐AM) prior to cell seeding (Figure 3a). Pre‐labeled HDFs were mixed with MCF‐7 cells at different ratios (MCF‐7: HDF = 3:1, 1:1, 1:3), together with a monoculture MCF‐7 control. During co‐culture, HDFs and MCF‐7 cells co‐aggregated within the same microwells to form integrated 3D spheroids, instead of remaining segregated in distinct regions without interaction. Interestingly, after 16 h incubation, the pre‐labeled HDFs exhibiting green fluorescence mostly accumulated in the central region, as further confirmed by a center‐peaked fluorescence profile (Figure 3b). On day 4, the initially labeled HDFs remained concentrated in the center of co‐culture spheroids (Figure S8). This aggregation pattern led to the formation of core‐shell co‐culture spheroids, consistent with previous reports [31].

To evaluate the impact of different cell ratios on early spheroid viability, live/dead staining was performed after two days of culture. Both monoculture MCF‐7 spheroids and co‐culture spheroids generated at different seeding ratios exhibited high viability with no noticeable differences (Figure 3c). Although co‐culture spheroids containing higher proportions of HDFs displayed irregular morphology on Day 1, this did not compromise their overall spheroid viability. These findings demonstrate that co‐culture spheroids can be successfully generated and maintained on the MEG platform, providing a reliable foundation for subsequent drug testing.

### 2.3 Gradient Validation and Spheroid Drug Responses on the MEG Platform

**FIGURE 4:**
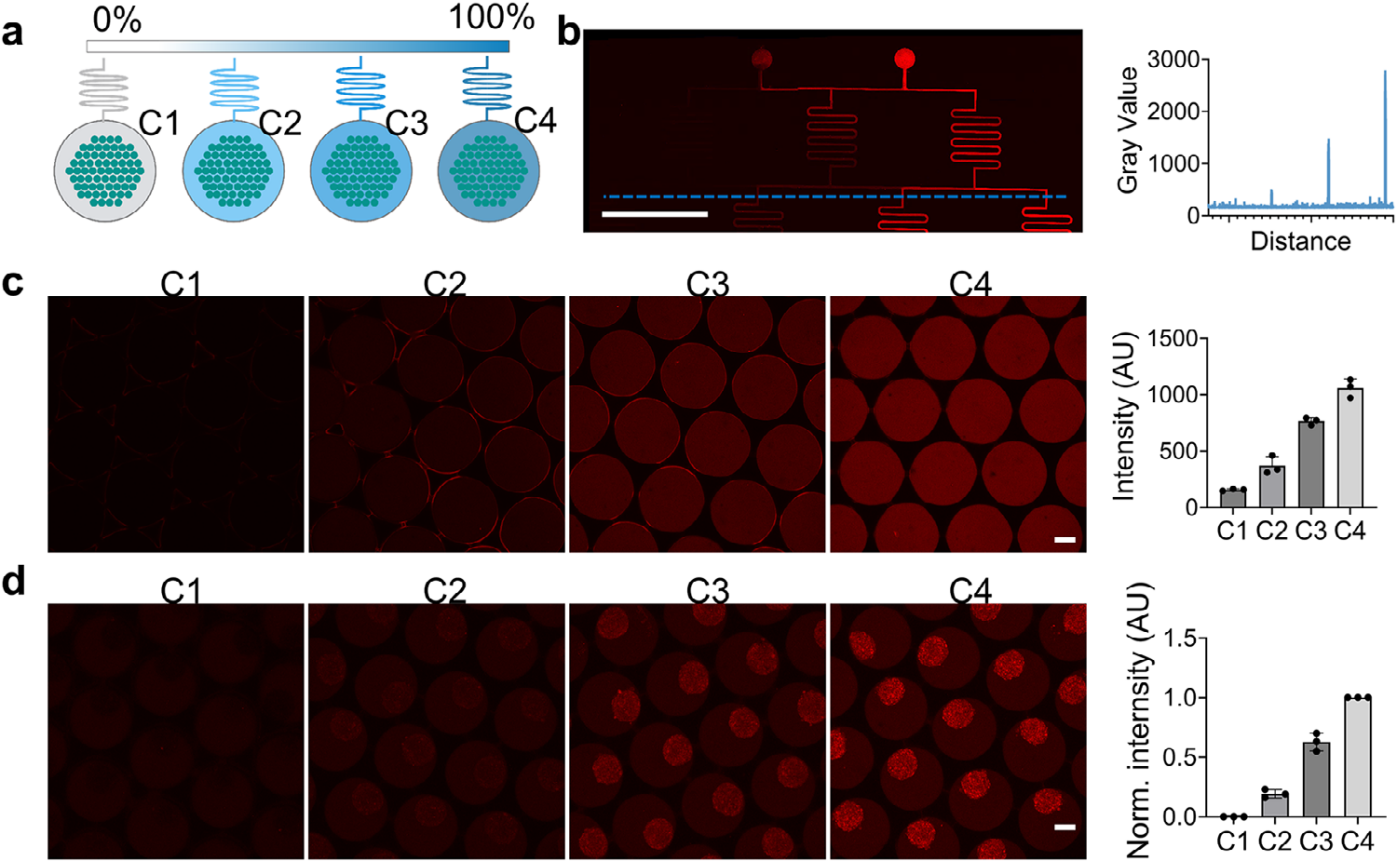
Gradient validation and doxorubicin (DOX) delivery analysis on the MEG platform. (a) Schematic of four downstream culture chambers, with a concentration gradient from C1 (lowest) to C4 (highest). (b) Fluorescence image of dextran in the Christmas tree mixing network after 10 h of perfusion, with line‐scan profile showing intensity distribution at the four branches. (c) Representative fluorescence images of dextran in microwell regions across C1–C4 chambers, with quantitative analysis of fluorescence intensity. (d) Representative DOX fluorescence images and quantitative analysis of DOX fluorescence intensity in MCF‐7 spheroids in C1‐C4 chambers under the gradient at 12 h. Fluorescence intensities were normalized to C1 (0%) and C4 (100%) for comparison. Scale bars: (b) 10 mm; (c,d) 100 µm. Data are presented as mean ± SD (n = 3 independent biological replicates). In (c) and (d), each data point represents the mean fluorescence intensity calculated from multiple fluorescence measurements (technical replicates).

The concentration gradient generated by the MEG platform was verified using dextran. Dextran was introduced from one inlet, while PBS was introduced from the other. The four downstream chambers were designated as C1‐C4, with C1 adjacent to the 0% inlet and C4 adjacent to the 100% inlet (Figure 4a). Fluorescence measurements across the gradient‐generator branches revealed a stable gradient distribution. Consistently, the fluorescence intensity profile showed three prominent peaks toward the higher‐concentration side, while the lowest‐concentration branch remained near baseline, validating gradient formation (Figure 4b). Fluorescence measured within the microwells of each chamber also increased from C1 to C4, indicating that the established gradient was effectively delivered to the culture chamber regions (Figure 4c).

To validate gradient delivery of drugs on the MEG platform, uniform MCF‐7 spheroids were pre‐exposed to a doxorubicin (DOX) gradient prior to long‐term treatment. A gradient was established by perfusing fresh medium and DOX‐containing medium through two inlets. After 12 h of perfusion, the fluorescence distribution of spheroids across chambers C1 to C4 followed the same trend observed during dextran validation. Spheroids in C4 displayed the highest fluorescence signal throughout the tumor spheroid structure, indicating efficient DOX penetration, while C2 and C3 exhibited intermediate intensities and C1 remained weak. Normalized fluorescence intensities increased progressively from C1 to C4, confirming the observed gradient distribution (Figure 4d). These results demonstrate that the MEG platform establishes a stable and controllable concentration gradient across chambers, which is effectively reflected in differential drug uptake within spheroids after 12 h of gradient DOX exposure.

We further validated the applicability of MEG platform for assessing tumor spheroid responses under dynamic drug flow. On separate platforms, monoculture MCF‐7 spheroids and co‐culture spheroids (MCF‐7: HDF = 1:1) were generated to evaluate their responses to gradient DOX treatment. After two days of culture, fresh medium and DOX‐containing medium (10 µm) were perfused through two inlets to establish a concentration gradient across the spheroid‐containing chambers. Previous studies have reported that spheroid size plays a critical role in determining drug responses [32]. In our preliminary validation, larger spheroids exhibited weaker DOX fluorescence intensity (Figure S9). Therefore, to ensure the accuracy of gradient drug testing, spheroid sizes were measured prior to drug loading. As shown in the violin plot (Figure S10), the mean diameters of all eight groups were consistently around 80 µm, confirming the reliability of subsequent drug response experiments.

**FIGURE 5:**
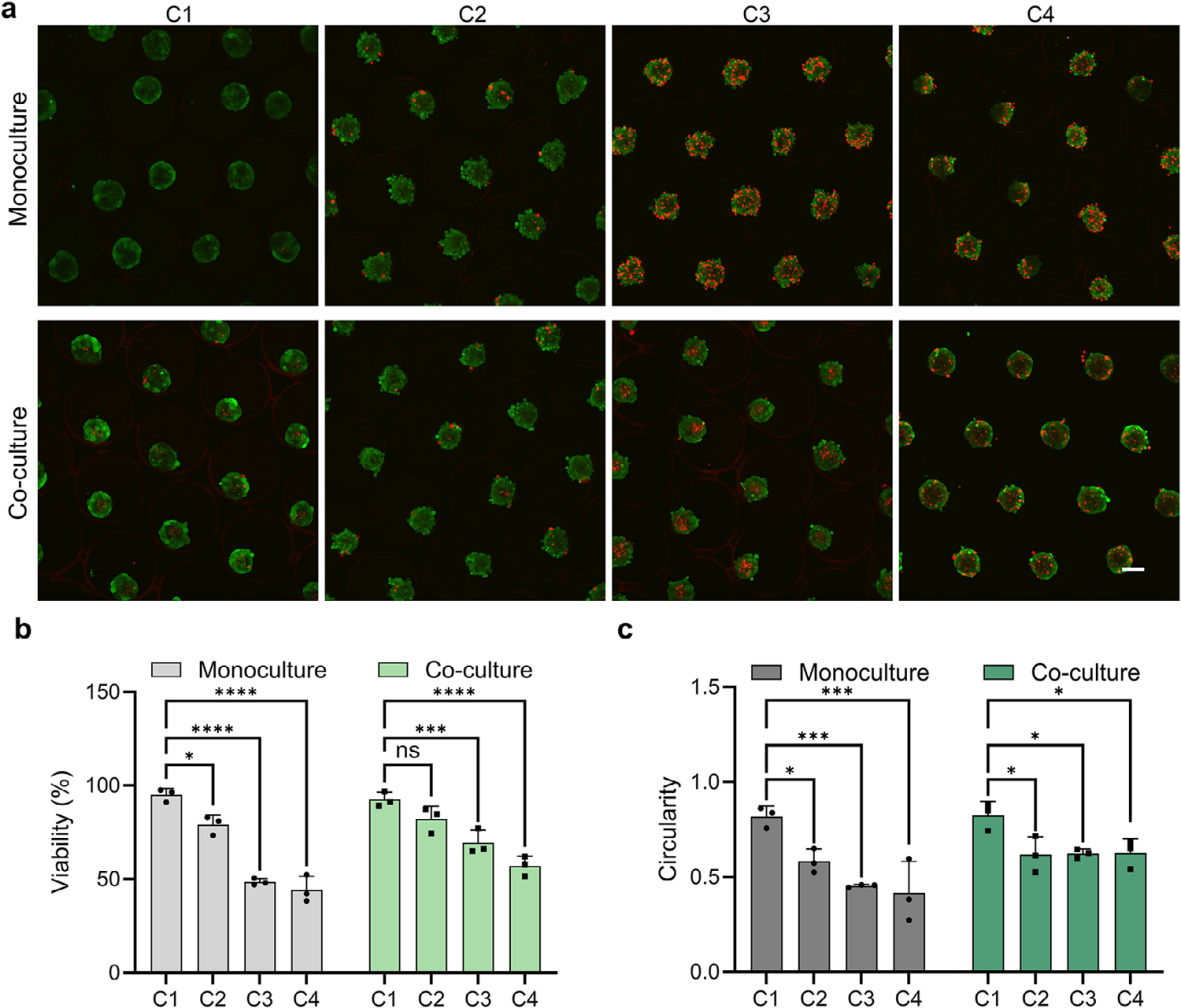
Drug responses of monoculture and co‐culture spheroids under gradient DOX treatment on the MEG platform. (a) Representative images of spheroid morphology and live/dead staining after 72 h of gradient DOX perfusion. C‐AM (live, green); EthD‐1 (dead, red). Quantitative analysis of spheroid viability (b) and circularity (c) after treatment. Scale bar: 100 µm. Data are presented as mean ± SD (n = 3 independent biological replicates). In (b) and (c), each data point represents the mean value of multiple spheroids (technical replicates). Statistical analysis was performed using two‐way ANOVA followed by Tukey's post‐hoc test. *p < 0.05, **p < 0.01, ***p < 0.001, ****p < 0.0001; NS, not significant.

The DOX concentrations across chambers C1–C4 were quantitatively calibrated as 0.29 ± 0.02, 2.13 ± 0.02, 6.25 ± 0.35, and 9.74 ± 0.41 µm, respectively, confirming the establishment of a stable concentration gradient within the MEG platform. Figure 5a shows live/dead staining images of spheroids after 72 h of dynamic drug treatment, revealing a progressive increase in cell death from C1 to C4 in both monoculture and co‐culture groups. In monoculture spheroids, C1 maintained a compact structure with smooth edges, whereas C2–C4 displayed varying degrees of edge irregularity. In the co‐culture group, cell death also increased from C1 to C4, although the spheroids retained relatively smoother edges than their monoculture counterparts.

Quantitative analysis of spheroid viability showed a progressive decrease with increasing DOX concentration after 72 h of dynamic drug treatment, consistent with the calibrated concentration gradient across chambers (C1–C4). In monoculture spheroids, viability decreased to approximately 44.3% in C4, with significant reductions observed in C2–C4 compared to C1. In contrast, co‐culture spheroids exhibited a more gradual decline, with no significant difference observed between C1 and C2, and retained approximately 57.0% viability in C4 (Figure 5b). Quantitative analysis showed significant reductions in circularity in C2–C4 compared with C1 in both culture models, with a greater reduction observed in monoculture spheroids. In the monoculture group, average circularity was 0.82 in the control chamber (C1) and progressively decreased to 0.57, 0.45, and 0.39 in C2–C4, respectively. In co‐culture spheroids, circularity values remained above 0.5 across all groups, indicating preserved structural integrity (Figure 5c). These results confirm that the MEG platform enables stable and controllable drug delivery across multiple spheroid groups. Under identical gradient conditions, co‐culture spheroids retained higher viability and exhibited a delayed onset of structural disruption, suggesting a potential fibroblast‐mediated protective effect.

## 3 Discussion

In this study, we developed an MEG platform that integrates a gradient generator with microwell array structures. This system enables drug gradient delivery while preserving the high‐throughput and size‐controllable advantages of microwell‐based spheroid culture. This MEG platform is compatible with both monoculture and tumor‐fibroblast co‐culture systems. MCF‐7 spheroids and co‐culture spheroids at different MCF‐7: HDF ratios maintained continuous growth. Notably, spheroids in the 1:3 co‐culture group exhibited reduced circularity on Day 1 but compacted into a round morphology by Day 2, resembling HDF monoculture spheroids. This observation is consistent with previous reports describing jagged and irregular boundaries of dermal spheroids during the early integration phase after seeding, which was attributed to the progressive cellular integration of fibroblasts [33]. Moreover, in this study, all co‐culture spheroids exhibited a core‐shell structure, with HDFs localized at the spheroid center. Such a pattern is supported by previous studies and is likely attributable to the differential cohesion between fibroblasts and tumor cells, as well as the faster aggregation dynamics of fibroblasts compared with tumor cells [31, 34, 35]. These observations highlight that the MEG platform not only supports stable spheroid growth but also captures early morphogenetic dynamics and spatial organization, underscoring its value for modeling tumor‐stroma interactions.

The dynamic gradient drug response of monoculture MCF‐7 spheroids and co‐culture spheroids (MCF‐7: HDF = 1:1) was also investigated on the MEG platform. The results confirmed the capability of the system to generate and maintain controlled drug concentration gradients. Furthermore, enhanced drug resistance of co‐culture spheroids was reproduced on this platform. Previous studies have reported that the introduction of fibroblasts increases spheroid compactness, thereby contributing to overall drug resistance [35, 36]. These results highlight the capability of the MEG platform to reproduce physiologically relevant drug resistance behaviors under controlled gradient conditions. Overall, compared with the conventional stepwise drug‐loading approach, the gradient platform provides continuous and dynamic drug exposure that is more physiologically relevant. By combining the high‐throughput capacity and structural stability of microwell assembly, it enables more reliable and effective drug testing. Moreover, applying gradient drug treatment to co‐culture 3D models highlights the potential of this platform as a reliable tool to investigate drug resistance in complex 3D culture systems.

Compared with existing microwell‐only or gradient‐only platforms, the MEG platform integrates high‐throughput spheroid formation with dynamic microenvironmental control. Conventional microwell systems enable the generation of uniform spheroids, but lack the ability to establish defined chemical gradients [37, 38, 39]. In contrast, gradient‐enabled microfluidic systems provide precise spatiotemporal control over soluble factors, yet are often limited in cell loading, long‐term perfusion, and spheroid throughput [19, 20, 21, 40, 41].

Other microfluidic spheroid platforms, such as droplet‐based and microstructure‐based systems, exhibit inherent trade‐offs between throughput, gradient stability, and culture duration [42, 43]. Droplet‐based microfluidic systems offer high throughput [44]. However, due to their discrete nature, establishing and maintaining stable and continuous concentration gradients typically require complex post‐processing, which makes precise control challenging and limits the ability to preserve ultrahigh throughput during dynamic gradient modulation [45, 46]. In contrast, microstructure‐based platforms provide superior spatial control, enabling uniform spheroid formation and localized microenvironment regulation [47]. Nevertheless, existing approaches that integrate microstructures with concentration gradients often face limitations in scalability, cell loading efficiency, and long‐term perfusion culture [19, 22, 48]. By integrating these complementary design features, the MEG platform enables simultaneous high‐throughput spheroid culture and spatially defined drug exposure. The system supports stable gradient perfusion during dynamic treatment and sustained spheroid culture for up to 7 days, and this unique integrated design distinguishes it from conventional microfluidic systems with limited functional integration, rendering it well‐suited for physiologically relevant 3D drug screening assays.

Despite the good performance of the MEG platform in dynamic drug treatment of tumor spheroids, certain limitations remain. From an engineering perspective, the integration of multiple functional modules increases fabrication complexity, while material‐related optical constraints associated with PDMS and device thickness may limit imaging compatibility. These challenges could be addressed by simplifying device architecture and optimizing material selection and optical design. Moreover, the platform currently employs an irreversible sealing strategy during perfusion, which ensures a stable gradient environment but sacrifices the flexibility of open‐well systems for spheroid retrieval and downstream analyses, potentially limiting more complex biological assays such as Western blotting and RNA sequencing. Future designs could explore re‐openable sealing structures or detachable modules to balance perfusion stability with operational flexibility [49].

At the design level, the limited number and configuration of gradient branches in our current platform restrict both the concentration resolution and combinatorial testing of multiple drugs. Strategies such as increasing the number of gradient branches or establishing orthogonal gradient systems may improve gradient resolution and enable combinatorial drug testing and support future scale‐up. On the biological side, the current system has only validated monoculture and two‐cell‐type co‐culture models, leaving substantial room for further exploration in terms of cellular model complexity. Incorporating stromal cell types (e.g., immune or endothelial cells) could enhance physiological relevance, while extending the systems to organoid models holds promise for modeling more complex tissue‐microenvironment interactions.

The MEG platform provides a robust framework for dynamic drug studies in both monoculture and co‐culture tumor spheroids. By integrating high‐throughput spheroid generation with controlled microenvironmental modulation, this platform expands the experimental capabilities of in vitro drug screening systems and enables the investigation of complex tissue‐level responses under defined microenvironmental conditions.

## 4 Conclusion

In this study, we established an MEG platform that integrates high‐throughput, size‐controllable spheroid formation with stable gradient perfusion. The platform reliably supports both monoculture MCF‐7 and MCF‐7/HDF co‐culture spheroids for at least seven days of culture, capturing key morphological behaviors and core‐shell organization. Gradient validation experiments and drug response assays confirmed that the MEG platform enables stable gradient delivery and reproducible spheroid responses, with co‐culture spheroids exhibiting increased drug resistance compared with monocultures. These results validate the robustness and applicability of the platform for controlled 3D spheroid‐based drug response studies.

The MEG platform integrates high‐throughput spheroid generation with dynamic microenvironmental control, thereby advancing the experimental capabilities of in vitro 3D drug screening models. This integrated design provides a scalable and physiologically relevant strategy for predictive drug evaluation, supporting the development of more sophisticated multicellular and organoid‐based systems.

## 5 Methods

### 5.1 Microfluidic Device Fabrication

Photolithography was used to fabricate the mold for the gradient generator using the MicroWriter ML3 system. After silanization, degassed PDMS (10:1 base‐to‐curing agent ratio; Motion) was poured onto the mold. After curing at 65 °C for at least 4 h, the PDMS replica was carefully peeled off the mold. Inlet and outlet ports, along with spheroid culture chambers, were then created using biopsy punches of appropriate diameters. Four PDMS microwell array sheets (e.g., 6 mm in diameter) were punched from a larger PDMS microwell slab, which was fabricated using a previously established method developed in our laboratory. Clean glass coverslips, gradient generator components, and PDMS microwell array sheets were treated in a plasma cleaner for 22 s prior to bonding. Based on the chamber layout of the gradient generator, PDMS microwell array sheets were sequentially bonded onto the glass coverslip, ensuring that each chamber corresponded to a single microwell array sheet. The gradient generator layer was then aligned and bonded on top. The assembled chip was placed on a 95 °C hotplate for 15 min to ensure stable bonding of all PDMS components to the glass substrate.

The microfluidic channels were designed in a serpentine configuration to enhance mixing efficiency and gradient generation. The total effective channel length, calculated along the serpentine path, was approximately 104.66 mm, with a channel width of 200 µm and a depth of 110 µm. The microwells had a diameter of 300 µm and a depth of 300 µm. The chamber volume was adjustable by varying the chamber diameter during fabrication, depending on experimental requirements. For example, a chamber with a diameter of 8 mm corresponded to a volume of approximately 200 µL.

### 5.2 Cell Culture

MCF‐7 cells, U87 MG cells, and human dermal fibroblasts (HDFs) were obtained from the American Type Culture Collection (ATCC). MCF‐7 and U87 MG cells were cultured in Dulbecco's Modified Eagle Medium (DMEM) supplemented with 10% fetal bovine serum (FBS) and 1% penicillin‐streptomycin (all from Thermo Fisher Scientific). HDFs were cultured in DMEM supplemented with 10% FBS, 4 mm L‐glutamine, and 20 mm HEPES (all from Thermo Fisher Scientific). All cell lines were maintained in a humidified incubator at 37 °C with 5% CO_2_. Cells were passaged or used for experiments when they reached 80–90% confluency. Cell lines were tested for mycoplasma contamination and confirmed to be negative.

### 5.3 Platform Treatment

Before cell seeding, two inlets were connected to a syringe pump, and the channel was flushed with deionized water (DI water) at a flow rate of 50 µL/min. After all air bubbles were removed from the channel, perfusion was stopped, and the tubing was cut, leaving approximately 4 cm of tubing attached to each inlet, which was clamped to prevent backflow. DI water was then removed from the spheroid culture chambers, and 150 µL of 1% (w/v) Pluronic F‐127 (PF‐127, Sigma‐Aldrich) solution was added for surface treatment. The chip was incubated for three successive 1 h periods, with bubble removal performed by gentle pipetting after each incubation, resulting in a total incubation time of about 3 h.

After incubation, the PF‐127 solution was removed from the culture chambers, followed by a rinse with 150 µL of phosphate‐buffered saline (PBS, Thermo Fisher Scientific). The chambers were then perfused with fresh cell culture medium using a syringe pump. Once the chambers were fully filled, perfusion was stopped, and the inlet tubing was cut again, leaving approximately 4 cm of tubing, and sealed with clamps.

### 5.4 Cell Loading and Spheroid Formation

Cells were harvested and resuspended at a density of 1 × 10^6^ cells/mL for seeding into microwells. Defined numbers of cells (e.g., 60, 180, or 300 cells per microwell) were obtained by adjusting the seeding concentrations accordingly. For co‐culture experiments, MCF‐7 cells and HDFs were mixed at defined ratios (3:1, 1:1, or 1:3) prior to seeding, with the total cell number per microwell kept constant. For specific groups designed to track HDF distribution, HDFs were pre‐labeled with Calcein‐AM (2 µm, Thermo Fisher Scientific) for 30 min at 37°C before mixing with MCF‐7 cells.

After preparation of the cell suspension, the culture medium in the chambers was gently removed and replaced with 150 µL of fresh cell suspension. If air bubbles were observed, they were carefully removed using gentle pipetting. Cell suspension was gently and uniformly mixed by pipetting immediately before loading to maintain a homogeneous cell density, and then rapidly transferred into the four chambers directly. After loading, the chamber was maintained horizontally and kept static for about 10 min to allow complete cell sedimentation. The chip was then incubated for 30 min to allow the cells to settle into the microwells by gravity. MCF‐7 cells spontaneously aggregated into compact spheroids under static culture.

Spheroid morphology and the spatial distribution of HDFs in co‐culture were imaged using a confocal microscope (FV3000s, Olympus). For spheroid formation efficiency analysis, spheroids formed at different initial cell seeding densities were imaged using a confocal microscope equipped with a live‐cell incubation system (Zeiss LSM 880). Imaging was performed at 10× magnification using the tile scan function to capture large‐area fields.

To visualize the 3D structure, spheroids were stained with CellMask Orange (Thermo Fisher Scientific) according to the manufacturer's protocol. For high‐resolution imaging of individual spheroids, samples were fixed with 4% paraformaldehyde, permeabilized with Triton X‐100 (Thermo Fisher Scientific), and stained with DAPI (1:1000) following standard protocols. Images were processed using Imaris software (version 9.9.1) under a licensed installation.

### 5.5 Sealing of Culture Chambers After Cell Loading

When gradient perfusion treatment is required, the culture chambers are sealed at the top in advance. PDMS was prepared at a 6:1 (base to curing agent) ratio, and 3 g of the mixture was degassed and sterilized by UV exposure for 30 min. After the cells had completely settled into the microwells, a thin ring of uncured PDMS was applied around the periphery of each chamber opening using a 10–20 µL pipette tip. A sterile circular glass coverslip was then gently placed on top of each chamber, resting on the PDMS ring to cover the opening. After all culture chambers were sealed in this manner, the device was placed in a 37 °C incubator for about 20 h to allow the PDMS to fully cure and form a tight seal between the coverslip and the chamber surface.

### 5.6 Gradient Generation and Characterization

To validate the gradient profile within the microfluidic chip, the sealed chip was perfused with dextran (Dextran, Tetramethylrhodamine, 10 000 MW, Neutral; 50 µg/mL in PBS; Thermo Fisher Scientific) introduced through one inlet, while PBS was introduced through the other inlet. The two solutions were perfused into the device using a syringe pump at a constant flow rate of 1 µL/min. After 10 h of continuous flow, fluorescence images were acquired from the culture chambers using a confocal microscope (FV3000s, Olympus). The fluorescence intensity across the chambers was quantified using ImageJ software to assess the gradient distribution. To further confirm drug gradient delivery in spheroids, after spheroid formation, 5 µm doxorubicin (DOX, Sigma‐Aldrich) was introduced through one inlet, while fresh medium was supplied through the other. The platform was perfused for 12 h at the same flow rate, and DOX distribution in MCF‐7 spheroids across different culture chambers was assessed by fluorescence imaging.

### 5.7 Drug Evaluation

After completion of spheroid formation under static culture, the sealed chip was connected to syringe pumps for perfusion‐based drug treatment. Culture medium containing 10 µm DOX was introduced into one inlet, while fresh medium was introduced into the other. The assembled chip was perfused at a constant flow rate of 1 µL/min, and the spheroids in each chamber were then exposed to the generated DOX gradient for 72 h under standard incubator conditions (37°C, 5% CO_2_).

To determine the concentration of DOX in each chamber, medium samples were collected after the system reached a stable exchange state. A standard calibration curve was established by measuring the fluorescence intensity of DOX solutions with a series of known concentrations. The fluorescence intensity of both standard solutions and chamber samples was measured under identical instrumental settings using a CLARIOstar Plus (BMG Labtech) microplate reader. The actual DOX concentration in each chamber was then calculated by interpolation from the standard calibration curve.

### 5.8 Live/Dead Assay

A live/dead staining solution was prepared in PBS containing 2 µm Calcein‐AM and 4 µm Ethidium Homodimer‐1 (EthD‐1; Thermo Fisher Scientific). The solution was introduced through the two inlets and perfused into the culture chambers at a flow rate of 20 µL/min for 30 min. After perfusion, samples on the platform were incubated under standard culture conditions for an additional 15 min, and the samples were subsequently imaged using a confocal microscope.

### 5.9 Statistical Analysis

Circularity was quantified as 4πA/P^2^, where A represents the spheroid area and P represents the perimeter, using ImageJ software. Statistical analysis was performed using GraphPad Prism 11 (version 11.0.0). Data are presented as mean ± standard deviation (SD), unless otherwise stated in the corresponding figure legends. No data transformation or normalization was applied unless explicitly specified. For comparisons involving more than two groups, statistical analysis was performed using two‐way ANOVA followed by Tukey's post‐hoc test. Differences were considered statistically significant at *p* < 0.05. For each condition, spheroids were randomly selected for quantitative analysis. The number of biological replicates (n) for each experiment is indicated in the corresponding figure legends. Experiments affected by accidental sample mixing or technical handling errors were excluded from analysis prior to statistical evaluation.

## Conflicts of Interest

The authors declare no conflicts of interest.

## Supporting information




**Supporting Information File 1**: smll73432‐sup‐0001‐SuppMat.docx.

**Supporting Information File 2**: smll73432‐sup‐0002‐FigureS1‐S11.zip.

## Data Availability

The data that support the findings of this study are available from the corresponding author upon reasonable request.
