# Supplementary material for: A Microwell Array Embedded Microfluidic Gradient Platform for Drug Screening on Tumor Spheroids
Source: Small. 2026 Apr 21;22(32):e14775. doi: 10.1002/smll.202514775 (PMC13244429; doi:10.1002/smll.202514775)
Supplement: Supplementary file 1 — Supporting Information File 1: smll73432‐sup‐0001‐SuppMat.docx. [file SMLL-22-e14775-s002.docx]

**Supporting Information**

**A Microwell Array Embedded Microfluidic Gradient Platform for Drug Screening on Tumor Spheroids**

Ling Liu^a, c^, Guoying Wang^b^ and Ming Li^a, c,^ *

1. *School of Engineering, Macquarie University, Sydney, NSW 2109, Australia*
2. *Macquarie Medical School, Faculty of Medicine, Health and Human Sciences, Macquarie University, Sydney, New South Wales 2109, Australia*
3. *School of Mechanical and Manufacturing Engineering, The University of New South Wales, Sydney, NSW, 2052 Australia*

E-mail: [ming.li3@unsw.edu.au](mailto:ming.li3@unsw.edu.au)

**
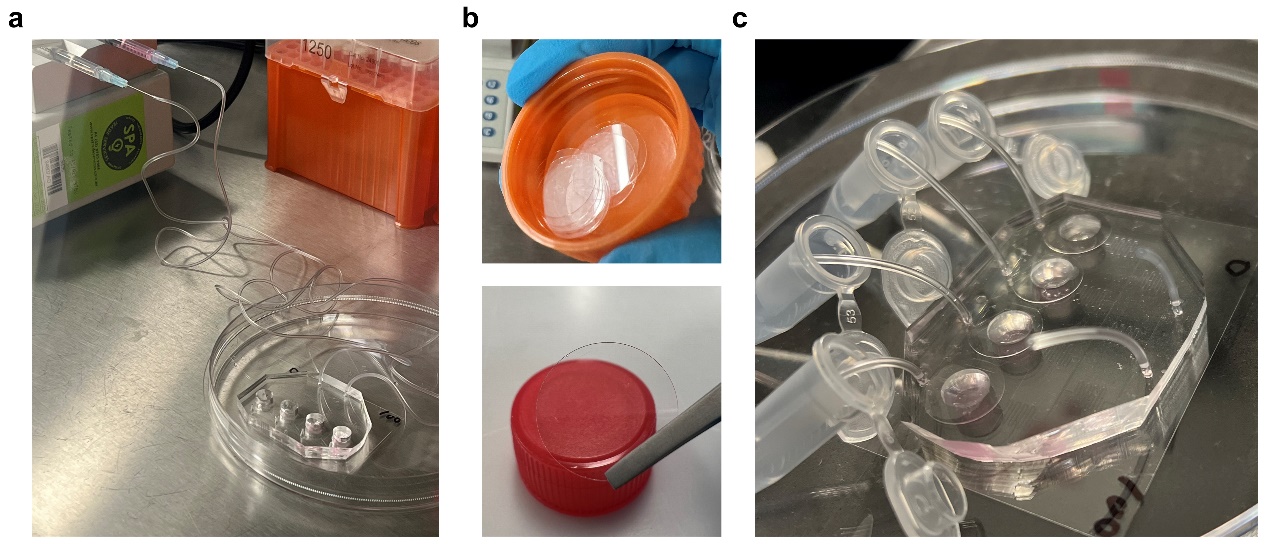
**

**Figure S1.** Overview of device setup and sealing for the MEG platform. (a) Photograph of the MEG chip connected to a syringe pump. (b) Photograph of the circular cover glass used for chamber sealing. (c) Example of a MEG chip after chamber sealing with the cover glass.


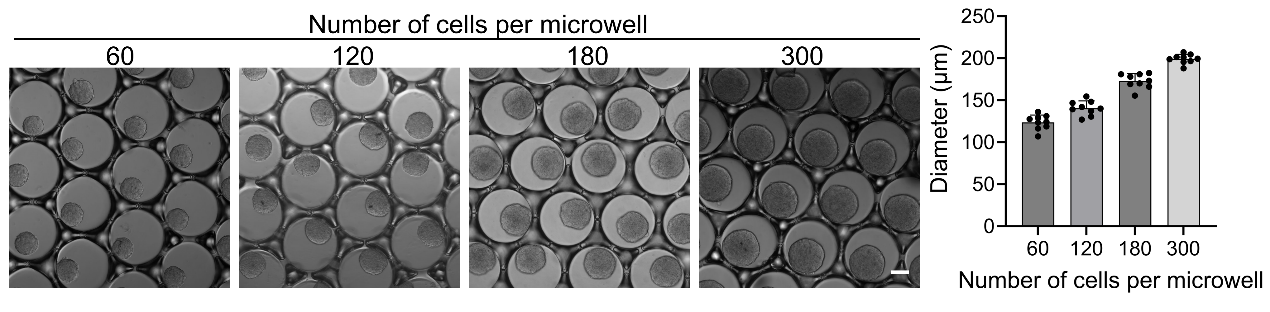


**Figure S2.** Morphology and diameter analysis of MCF-7 spheroids on Day 4 at different initial seeding densities. Scale bar: 100 µm. Data are presented as mean ± SD (n = 3 independent biological replicates). Each dot represents the average spheroid diameter in one chamber (three chambers per condition).


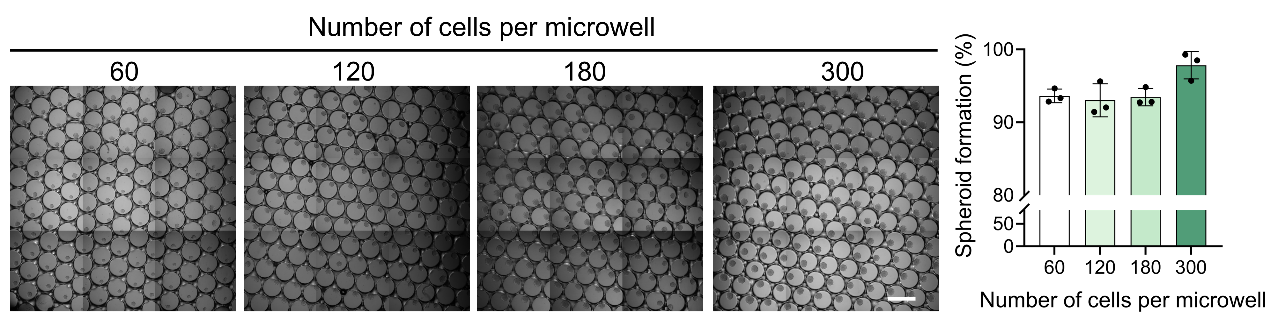


**Figure S3**. Representative images and quantitative analysis of spheroid formation efficiency at different initial seeding densities. Scale bar: 500 µm. Data are presented as mean ± SD (n = 3 independent biological replicates).


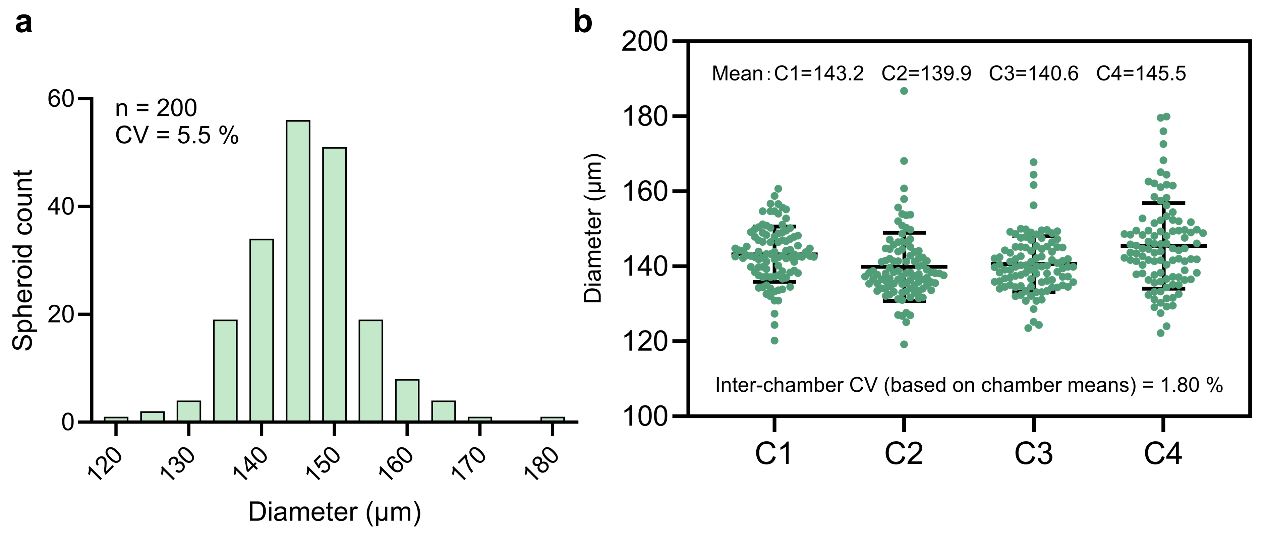


**Figure S4**. Size distribution and reproducibility of spheroids generated by the integrated microwell platform. (a) Histogram showing the diameter distribution of spheroids generated within a single representative chamber, demonstrating excellent size uniformity (CV = 5.5%, n = 200 spheroids). (b) Size distribution of spheroids across four independent chambers (n = 100 spheroids per chamber). The inter-chamber CV calculated from the four chamber means was 1.80%, confirming high reproducibility and consistent size control across the platform.


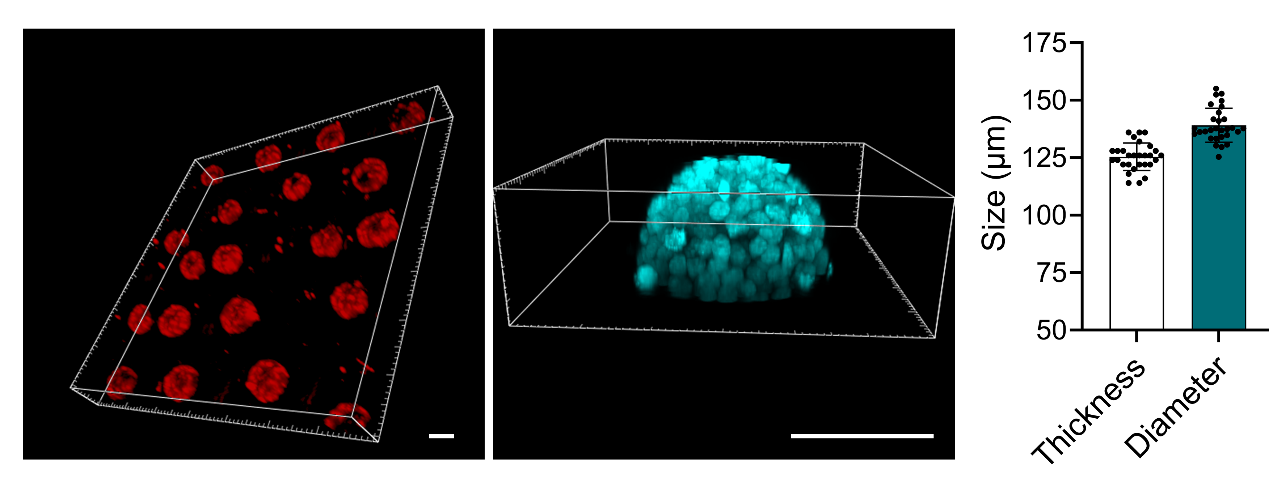


**Figure S5.** Three-dimensional characterization of spheroid morphology by confocal Z-stack imaging. Representative confocal Z-stack images at different scales are shown, including a wide-field view of multiple spheroids (CellMask™ Orange) and a single-spheroid reconstruction (DAPI). Scale bar: 100 µm. Quantitative analysis of spheroid thickness and diameter is shown on the right. The comparable thickness and diameter values indicate a nearly spherical morphology and high structural homogeneity. Data are presented as mean ± SD (n = 30 spheroids from 3 independent experiments), with individual data points shown.


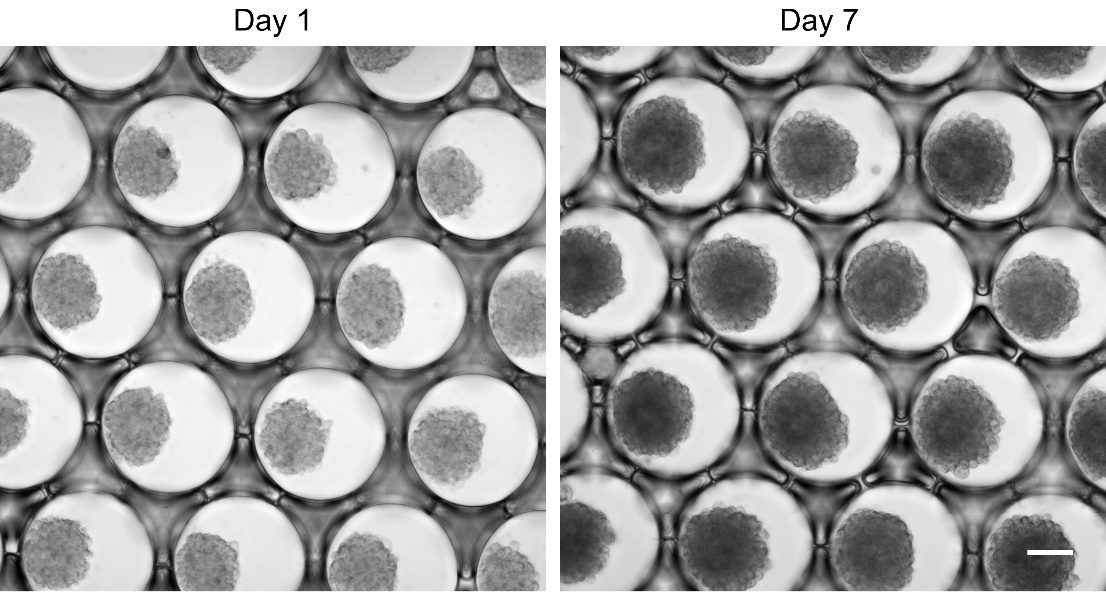


**Figure S6.** Representative bright-field images of U87 MG spheroids formed in 300 µm × 300 µm microwells after 1 day and 7 days of culture, demonstrating the applicability of the platform to different cell types. Scale bar: 100 µm.


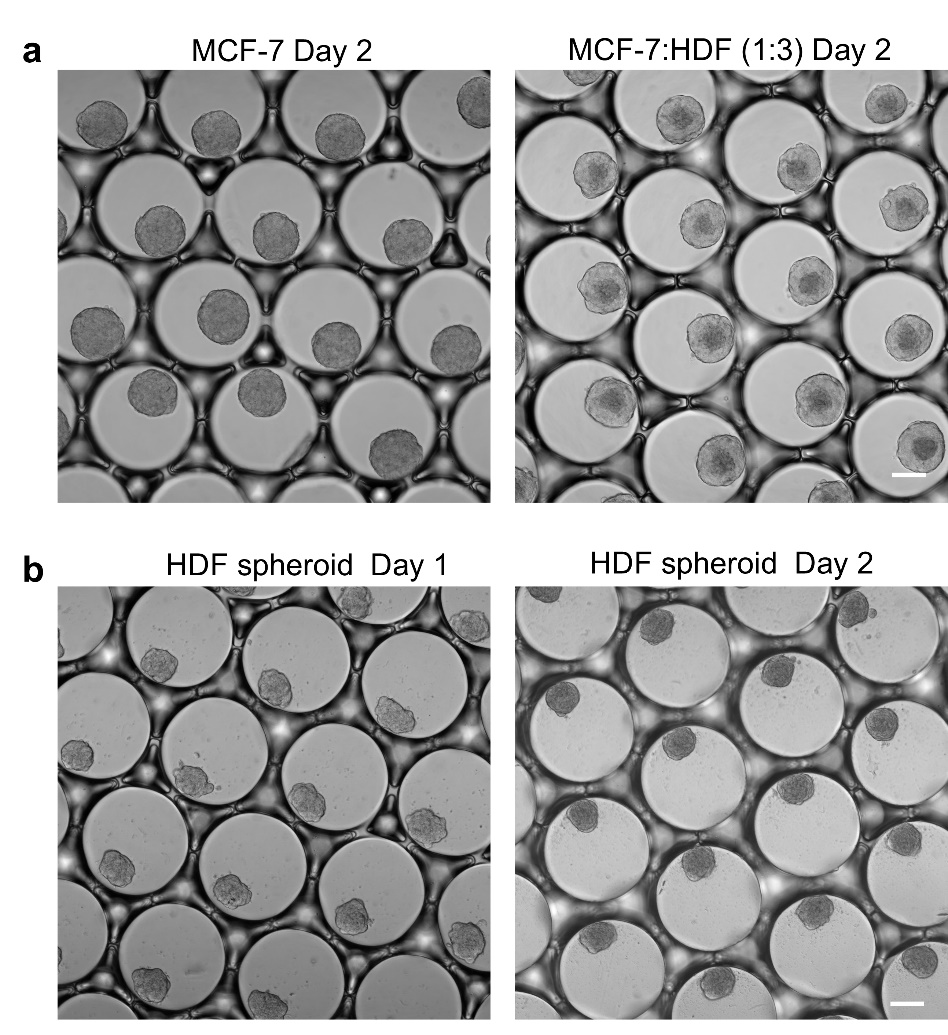


**Figure S7.** (a) Representative bright-field images of control monoculture spheroids and MCF-7: HDF (1:3) co-culture spheroids on Day 2. (b) Morphological changes of HDF spheroids from Day 1 to Day 2, showing a more spherical morphology on Day 2. Scale bar: 100 µm.


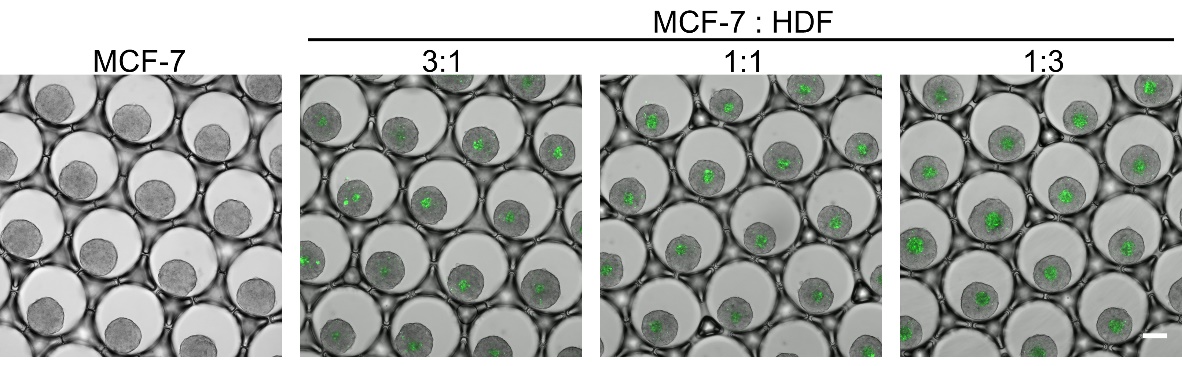


**Figure S8.** Morphology of co-culture spheroids on Day 4 with pre-labeled HDF at varying MCF-7: HDF ratios, showing residual fluorescence in the spheroid core. Monoculture spheroids are included as controls. Scale bar: 100 µm.


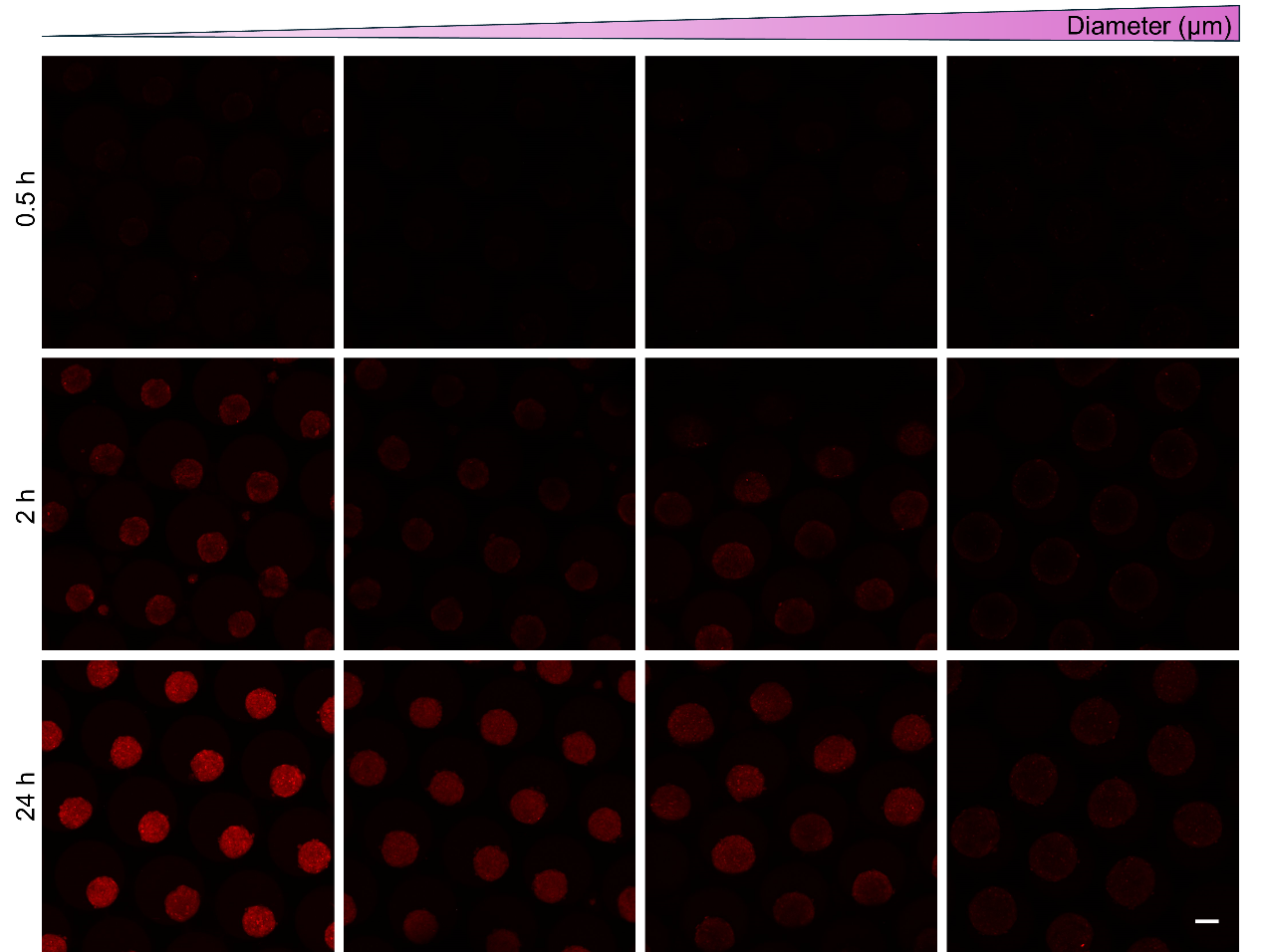


**Figure S9**. Doxorubicin distribution in MCF-7 spheroids of different diameters after 5 µM treatment for 0.5, 2, and 24 h. Scale bar: 100 µm.


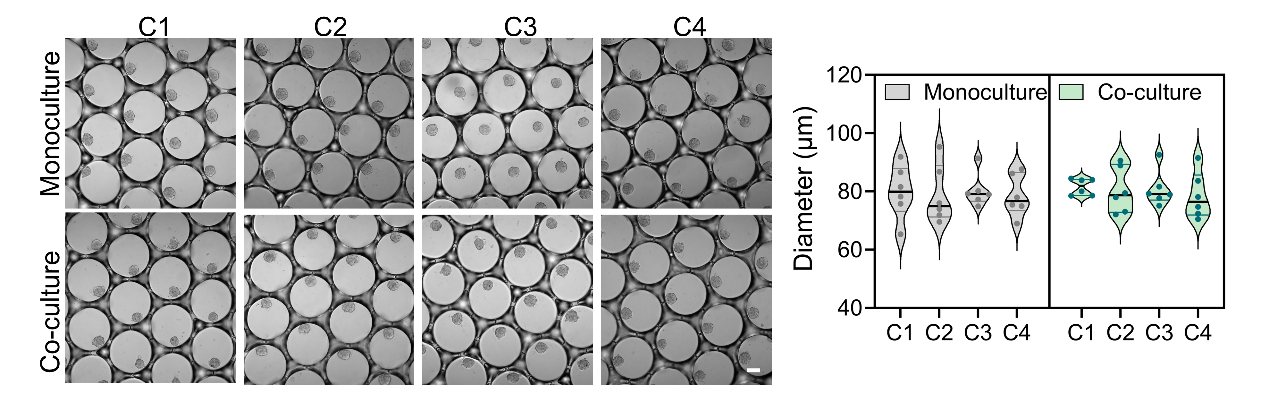


**Figure S10.** Bright-field images and diameter analysis of MCF-7 monoculture spheroids and MCF-7: HDF (1:1) co-culture spheroids prior to DOX treatment. Spheroids across chambers showed consistent size distributions, enabling matched comparison. Scale bar: 100 µm. Data are shown as violin plots with median and quartiles (n = 3 independent biological replicates).


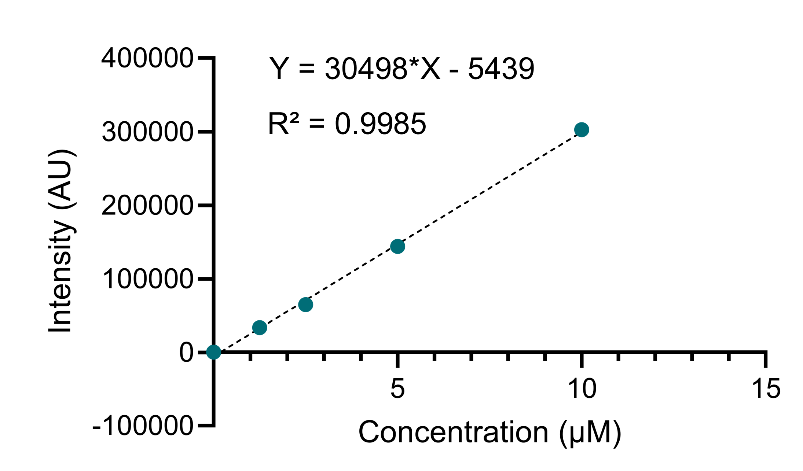


**Figure S11.** Representative calibration curve of DOX concentration versus fluorescence intensity.
