# Supplementary figures and images for: A Microwell Array Embedded Microfluidic Gradient Platform for Drug Screening on Tumor Spheroids
Source: Small. 2026 Apr 21;22(32):e14775. doi: 10.1002/smll.202514775 (PMC13244429; doi:10.1002/smll.202514775)

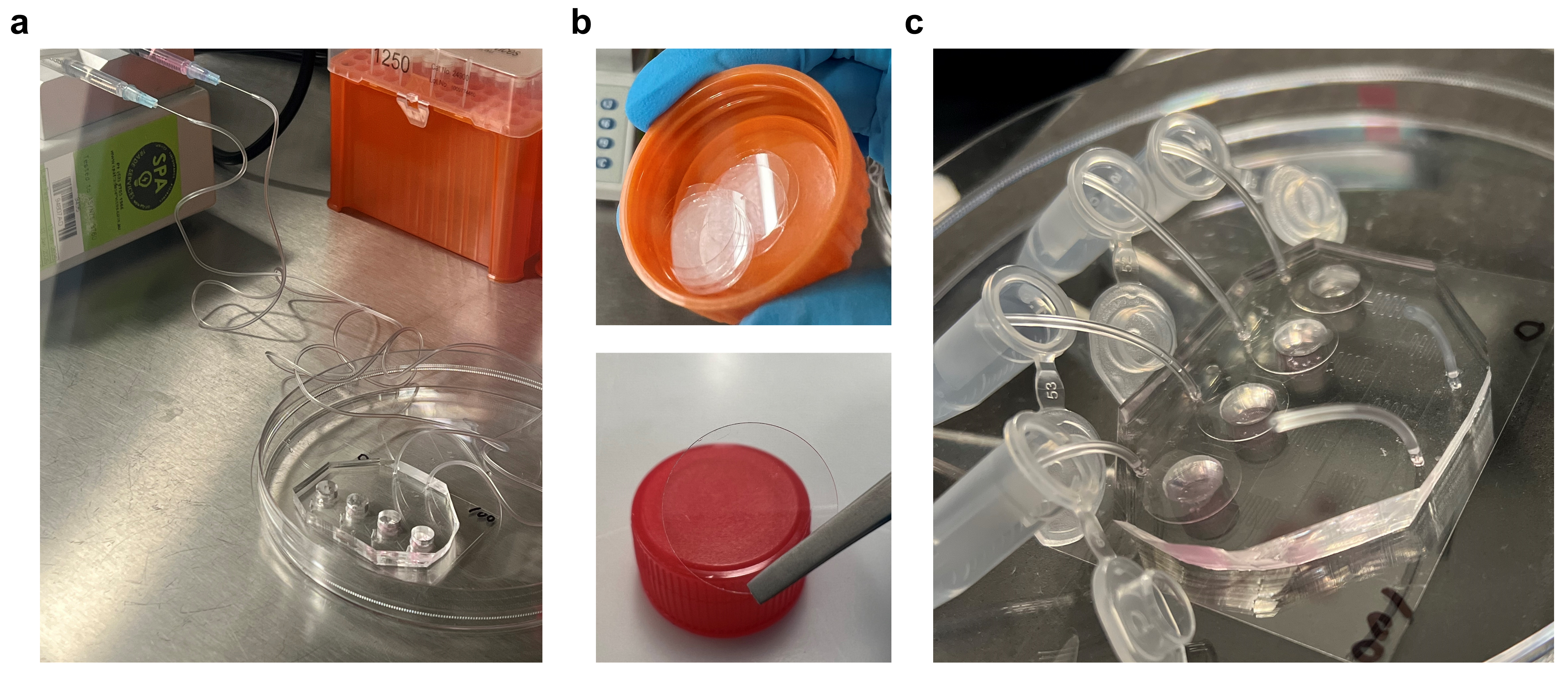

Supplement: Supplementary file 2 — Supporting Information File 2: smll73432‐sup‐0002‐FigureS1‐S11.zip. [file SMLL-22-e14775-s001.zip › Figure S1.tif]

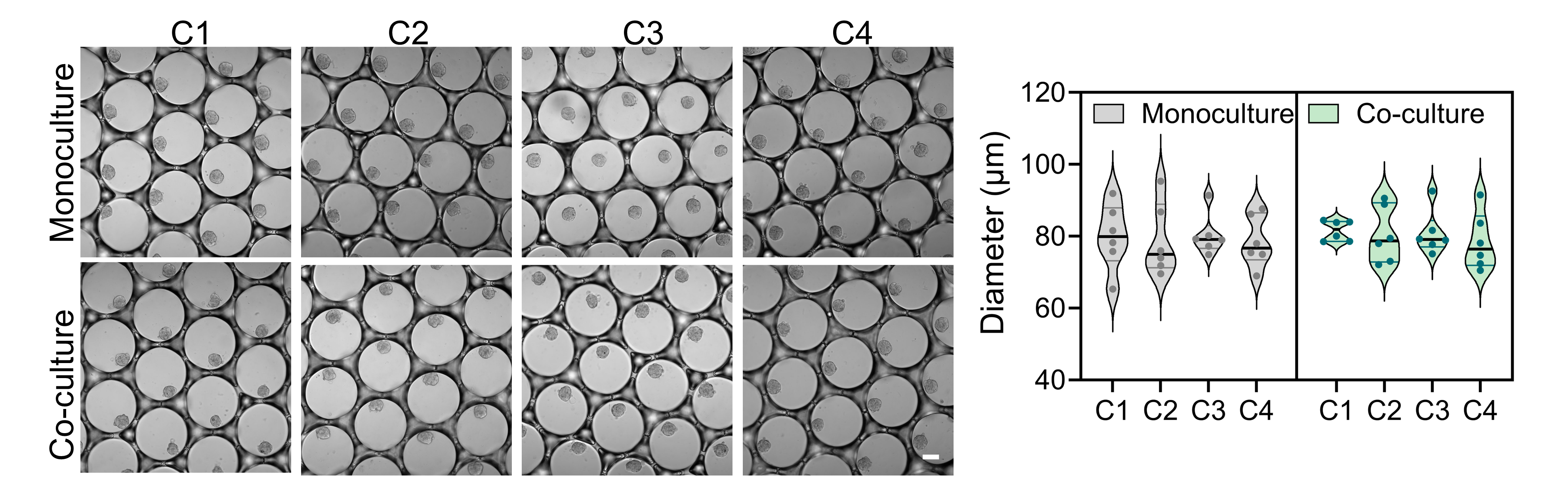

Supplement: Supplementary file 2 — Supporting Information File 2: smll73432‐sup‐0002‐FigureS1‐S11.zip. [file SMLL-22-e14775-s001.zip › Figure S10.tif]

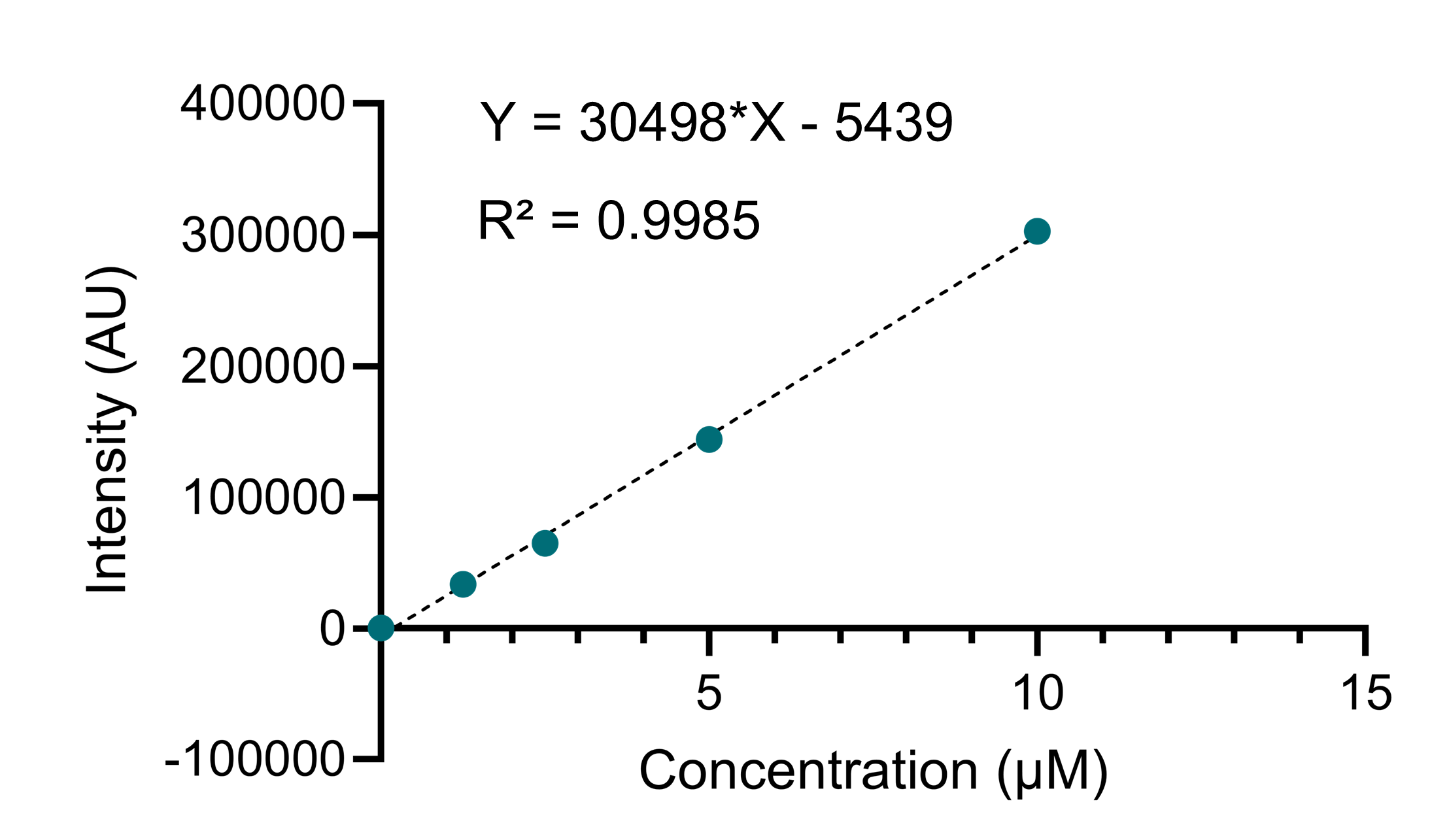

Supplement: Supplementary file 2 — Supporting Information File 2: smll73432‐sup‐0002‐FigureS1‐S11.zip. [file SMLL-22-e14775-s001.zip › Figure S11.tif]

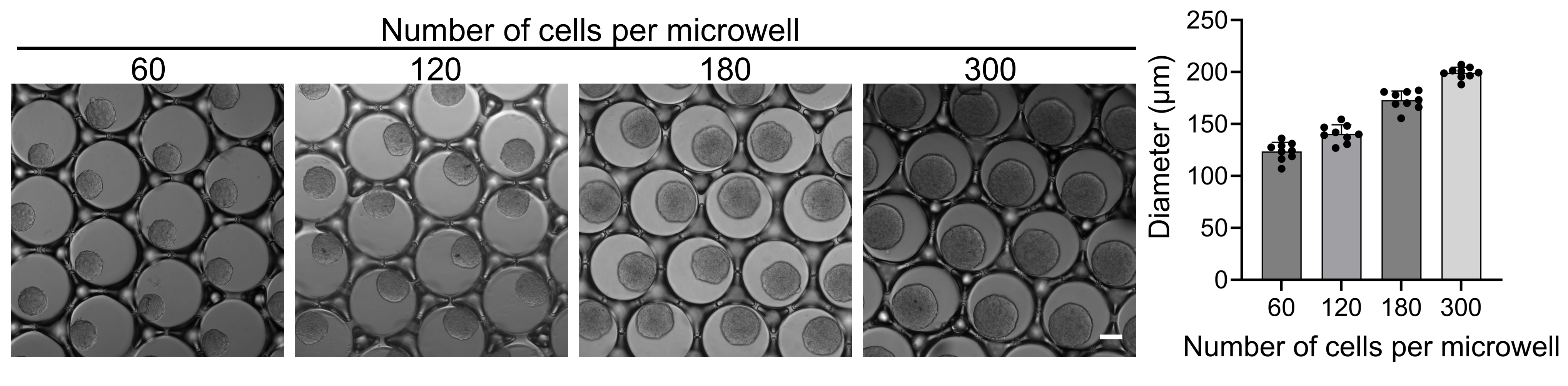

Supplement: Supplementary file 2 — Supporting Information File 2: smll73432‐sup‐0002‐FigureS1‐S11.zip. [file SMLL-22-e14775-s001.zip › Figure S2.tif]

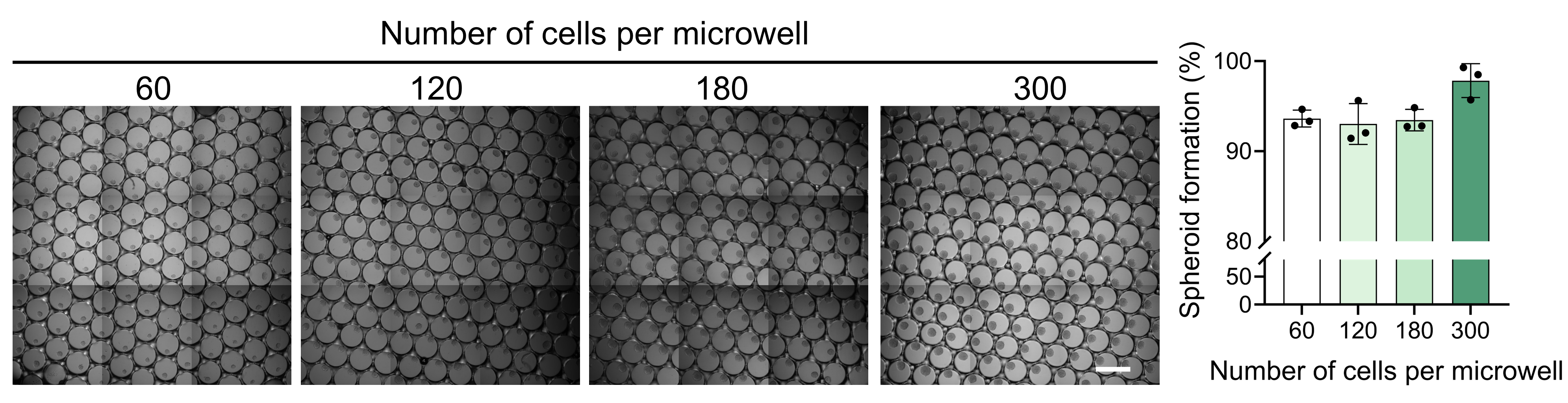

Supplement: Supplementary file 2 — Supporting Information File 2: smll73432‐sup‐0002‐FigureS1‐S11.zip. [file SMLL-22-e14775-s001.zip › Figure S3.tif]

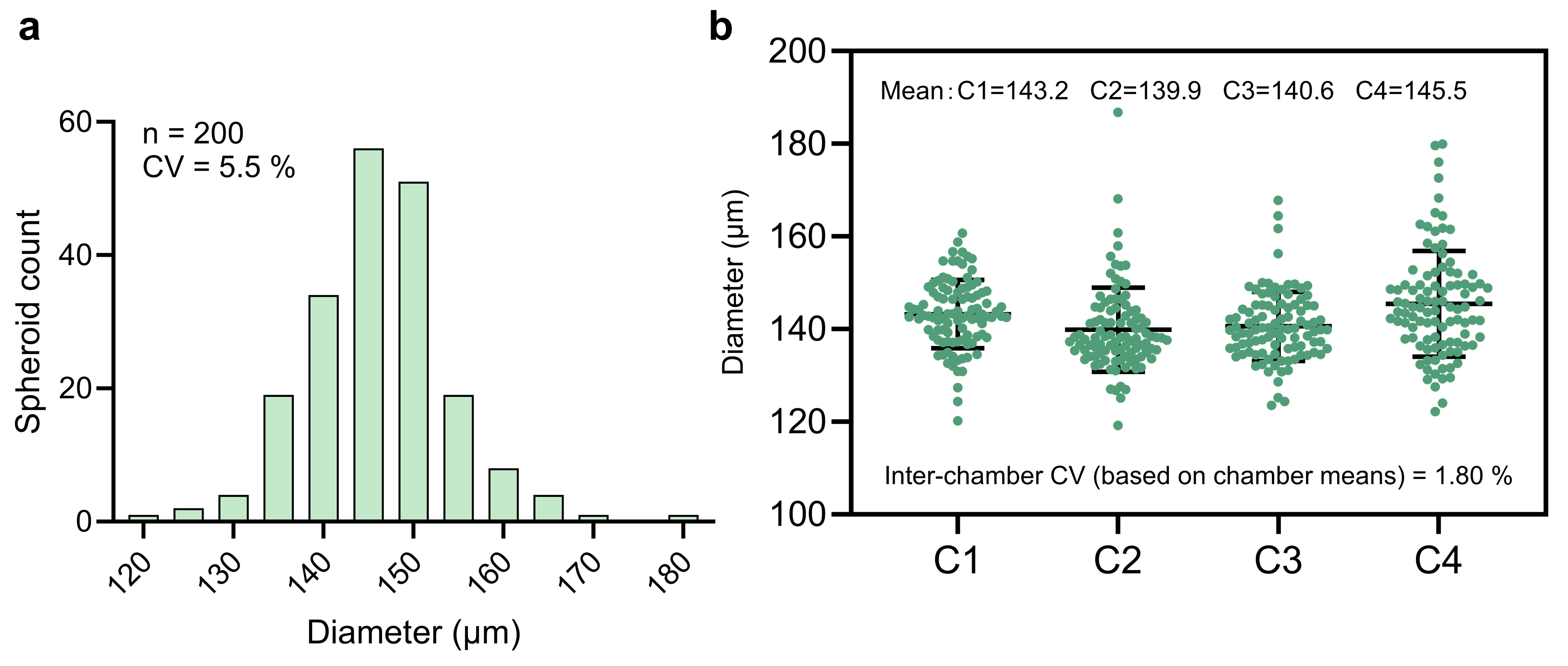

Supplement: Supplementary file 2 — Supporting Information File 2: smll73432‐sup‐0002‐FigureS1‐S11.zip. [file SMLL-22-e14775-s001.zip › Figure S4.tif]

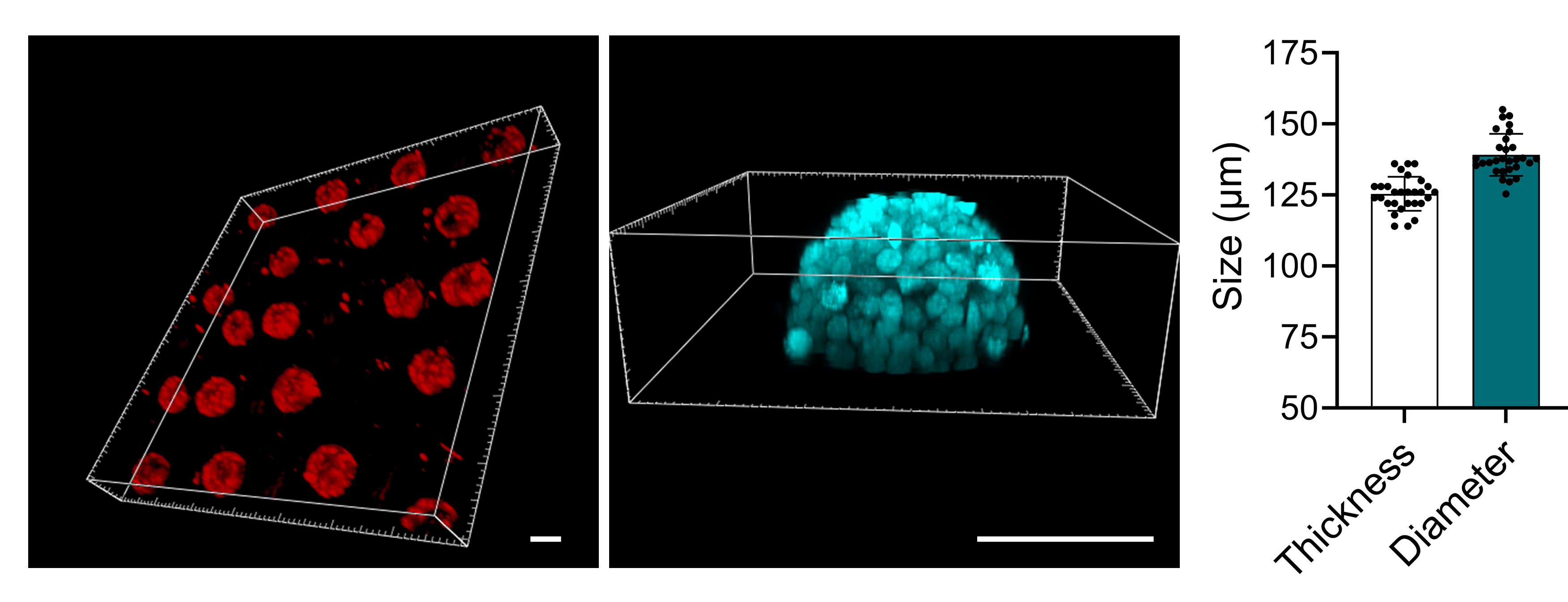

Supplement: Supplementary file 2 — Supporting Information File 2: smll73432‐sup‐0002‐FigureS1‐S11.zip. [file SMLL-22-e14775-s001.zip › Figure S5.tif]

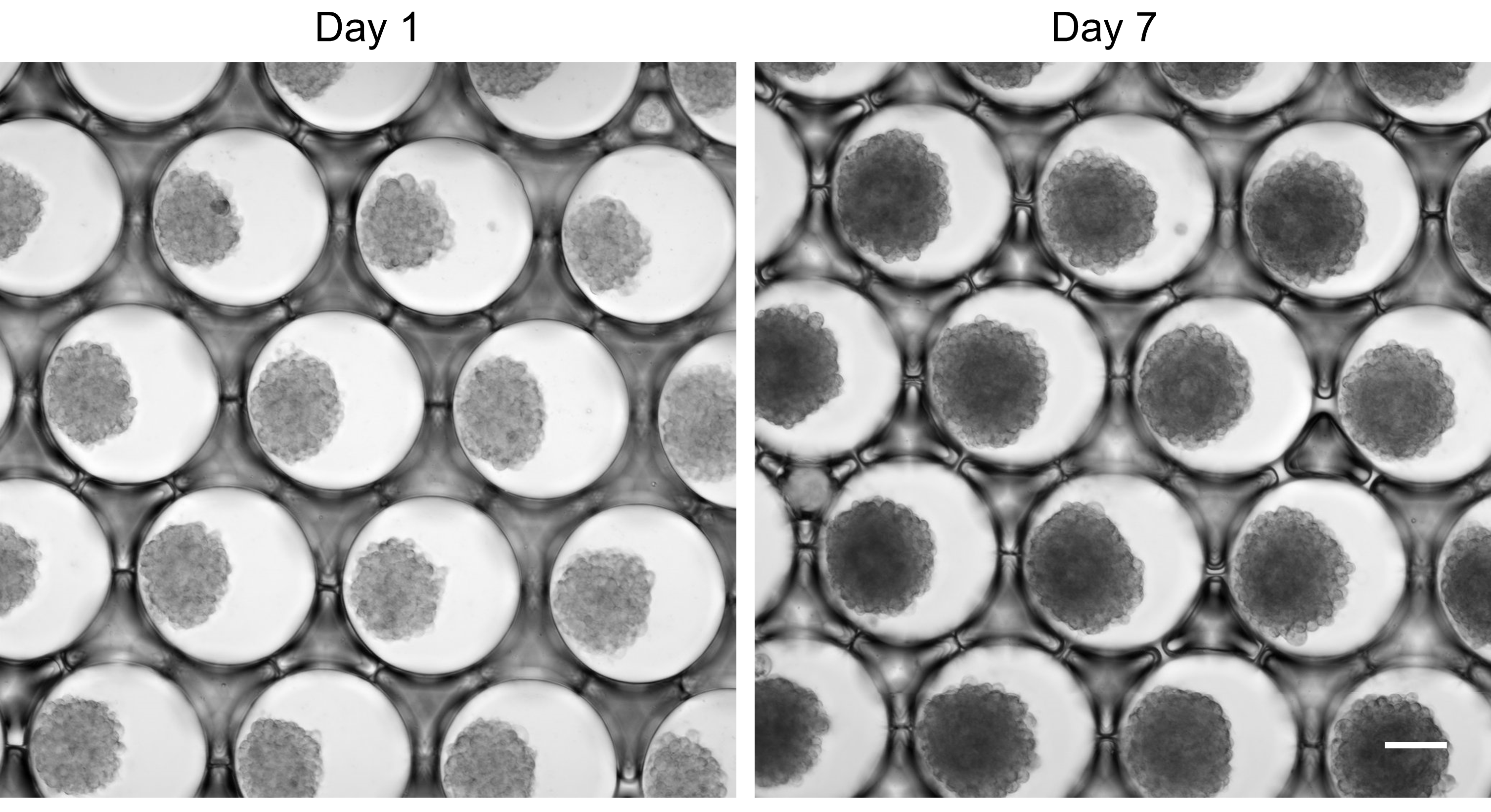

Supplement: Supplementary file 2 — Supporting Information File 2: smll73432‐sup‐0002‐FigureS1‐S11.zip. [file SMLL-22-e14775-s001.zip › Figure S6.tif]

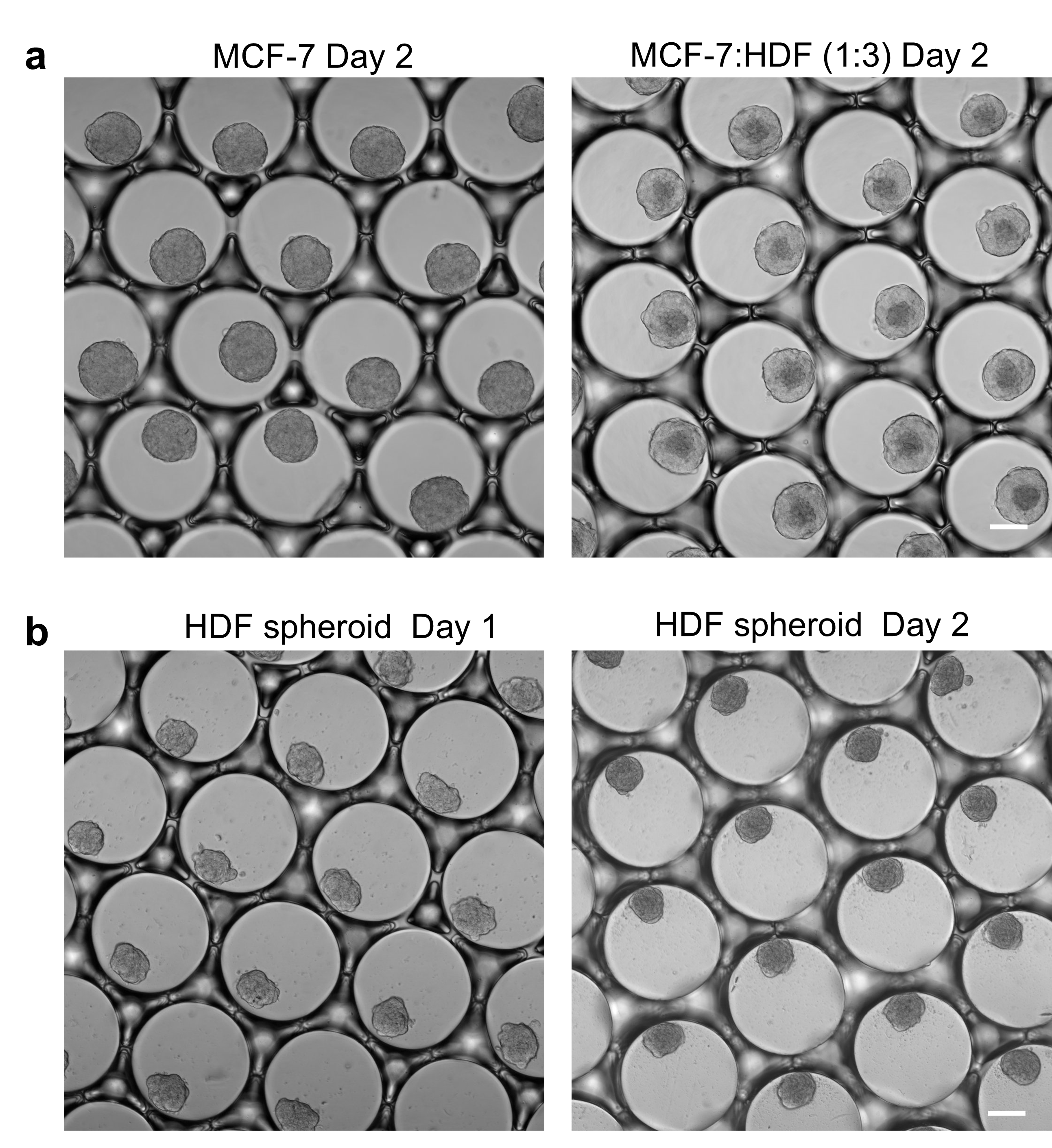

Supplement: Supplementary file 2 — Supporting Information File 2: smll73432‐sup‐0002‐FigureS1‐S11.zip. [file SMLL-22-e14775-s001.zip › Figure S7.tif]

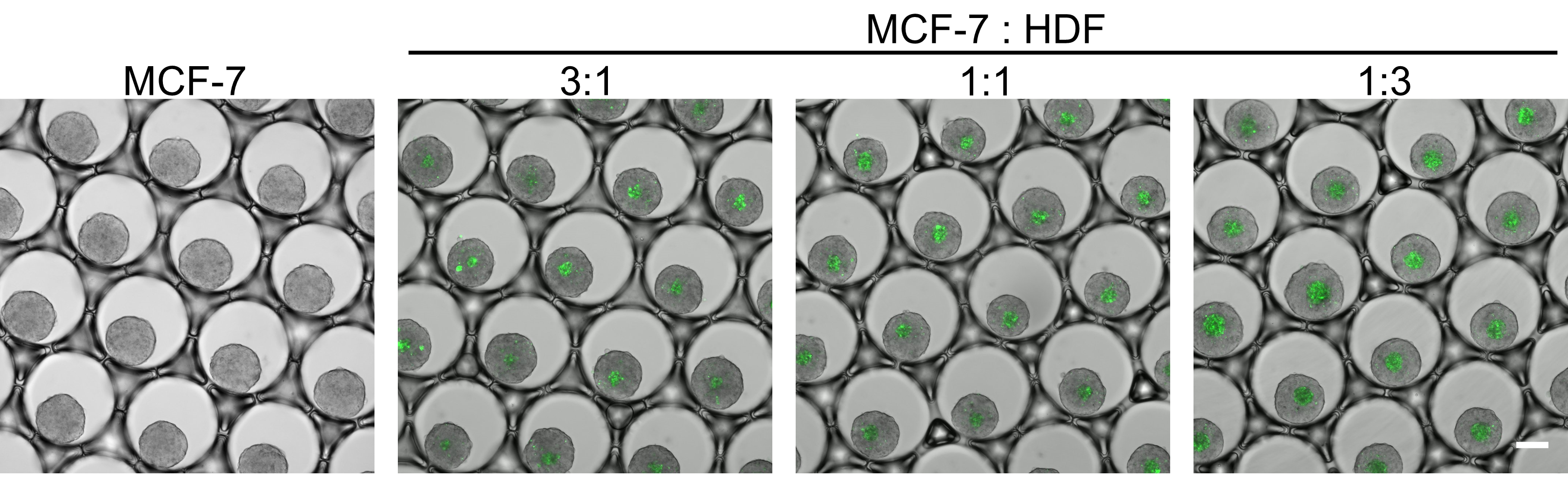

Supplement: Supplementary file 2 — Supporting Information File 2: smll73432‐sup‐0002‐FigureS1‐S11.zip. [file SMLL-22-e14775-s001.zip › Figure S8.tif]

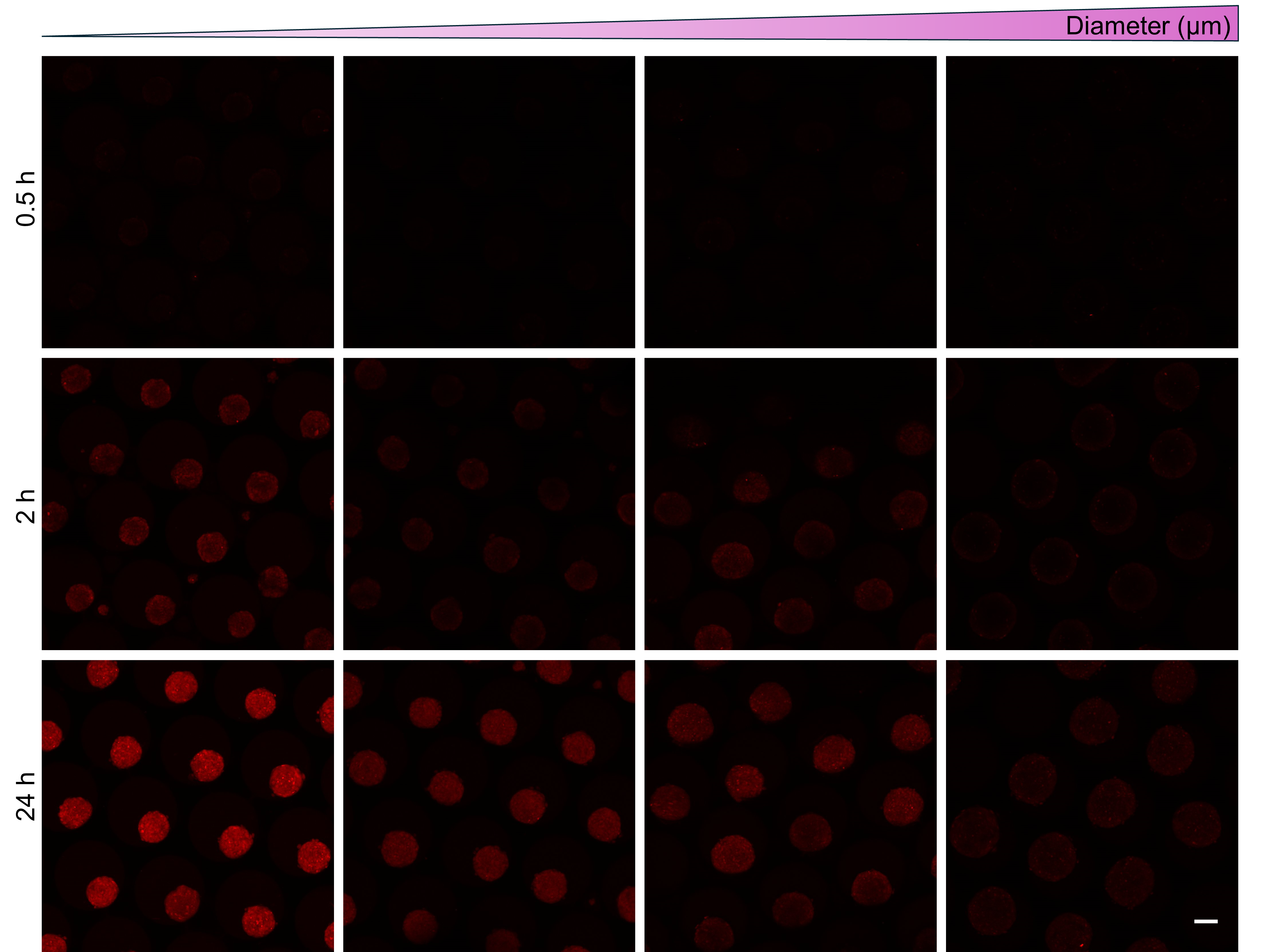

Supplement: Supplementary file 2 — Supporting Information File 2: smll73432‐sup‐0002‐FigureS1‐S11.zip. [file SMLL-22-e14775-s001.zip › Figure S9.tif]
